# Precancerous Stem Cells Have the Potential for both Benign and Malignant Differentiation

**DOI:** 10.1371/journal.pone.0000293

**Published:** 2007-03-14

**Authors:** Li Chen, Rulong Shen, Yin Ye, Xin-An Pu, Xingluo Liu, Wenrui Duan, Jing Wen, Jason Zimmerer, Ying Wang, Yan Liu, Larry C. Lasky, Nyla A. Heerema, Danilo Perrotti, Keiko Ozato, Satomi Kuramochi-Miyagawa, Toru Nakano, Allen J. Yates, William E. Carson III, Haifan Lin, Sanford H. Barsky, Jian-Xin Gao

**Affiliations:** 1 Department of Pathology, Ohio State University Medical Center, Columbus, Ohio, United States of America; 2 Comprehensive Cancer Center, Ohio State University Medical Center, Columbus, Ohio, United States of America; 3 Center for Molecular Neurobiology, Ohio State University Medical Center, Columbus, Ohio, United States of America; 4 Center for Stem Cell and Regenerative Medicine, Cleveland, Ohio, United States of America; 5 Yale Stem Cell Center, Yale University School of Medicine, New Haven, Connecticut, United States of America; 6 Laboratory of Molecular Growth Regulation, National Institute of Child Health and Human Development, National Institutes of Health, Bethesda, Maryland, United States of America; 7 Department of Molecular Cell Biology, Research Institute for Microbial Diseases, Osaka University, Osaka, Japan; University of Hong Kong, China

## Abstract

Cancer stem cells (CSCs) have been identified in hematopoietic and solid tumors. However, their precursors—namely, precancerous stem cells (pCSCs) —have not been characterized. Here we experimentally define the pCSCs that have the potential for both benign and malignant differentiation, depending on environmental cues. While clonal pCSCs can develop into various types of tissue cells in immunocompetent mice without developing into cancer, they often develop, however, into leukemic or solid cancers composed of various types of cancer cells in immunodeficient mice. The progress of the pCSCs to cancers is associated with the up-regulation of c-kit and Sca-1, as well as with lineage markers. Mechanistically, the pCSCs are regulated by the PIWI/AGO family gene called *piwil2*. Our results provide clear evidence that a single clone of pCSCs has the potential for both benign and malignant differentiation, depending on the environmental cues. We anticipate pCSCs to be a novel target for the early detection, prevention, and therapy of cancers.

## Introduction

A variety of cancers can arise from reversible premalignant stages of hyperplasia and dysplasia, which might progress to primary and invasive tumors. Recent evidence indicates that the process is initiated by the cancer stem cells (CSCs) [1]–[3]. While CSCs have been identified in the hematopoietic and solid cancers [4]–[9], the mechanisms underlying CSC derivation are largely unknown. CSCs may originate from a stem or progenitor cell through a precancerous stage, during which stem cells are hierarchically disturbed in their genetic program of self-renewal by environmental insults [1], [6], [10], whereas the progenitor cells may acquire the properties of stem cells [11]. Thus, whether or not pCSCs exist and how they develop into cancer cells are critical issues for cancer stem cell biology.

While investigating tumor incidence in mice with the targeted mutation of p53 and stat-1 genes [12], [13], we found that a mouse developed dendritic cell (DC)-like leukemia, which was characterized by massive infiltration of CD11c^+^CD8α^+^ DCs in spleen, lymph nodes, and liver [14]. After cloning DC-like lines from the spleen of this leukemic mouse [14], 3 of 25 clones (designated as 2C4, 3B5C and 3B6C) expressed neither hematopoietic and lineage (Lin) markers nor hematopoietic stem cell (HSC) markers (CD45^−^c-kit^−^Sca-1^−^Lin^−^). These cells have the potential for both benign and malignant differentiation, and their fate appears to be determined by environmental cues. Since they have the properties of both normal stem cells and CSCs, we use the term pCSCs to recognize their hybrid-like characteristics. The progression of pCSCs to cancer cells is associated with up-regulation of c-kit and Sca-1, as well as with lineage markers. Mechanistically, their expansion is regulated by a PIWI/AGO gene *piwil2* (alias *mili* in mouse and *hili* in human) [15], [16]. These findings will help us develop a novel strategy for the early detection, prevention, and treatment of cancers.

## Results

### 1. pCSCs exhibit stem-like cell phenotype

**Figure 1:**
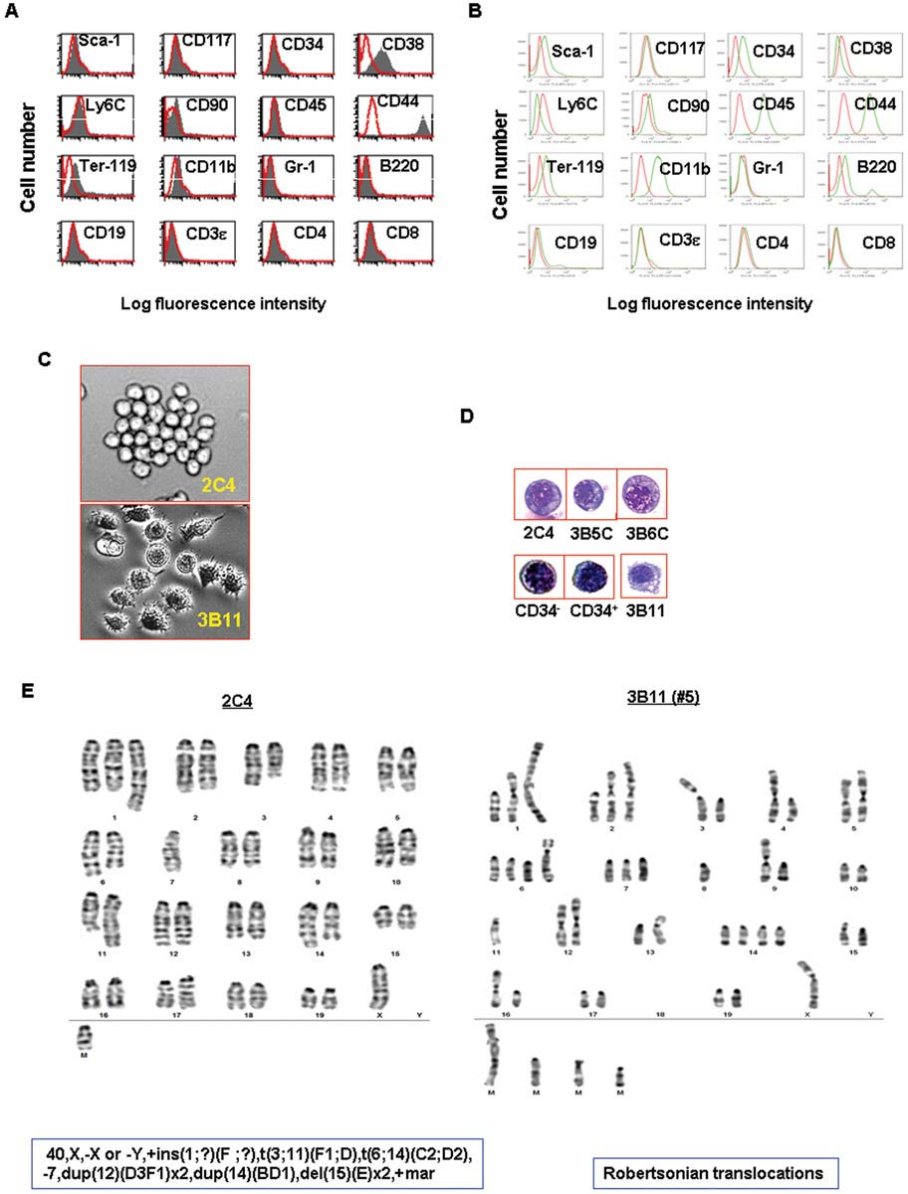
Phenotypical and cytological characterization of pCSCs. A & B, The phenotype of pCSC clones (A) and monocytic cell clones (B) from the same mouse (14): the data shown are a representative of pCSC (2C4; A) and monocytic clones (3B11; B). The phenotype of the 2C4 clone was similar to 3B5C and 3B6C clones (not shown). Red histograms represent isotype mAbs (A & B), and black (A) or green (B) histograms represent specific mAbs. C, Phase contrast micrographs: a representative (2C4) of 3 pCSC clones (top panel) and differentiated DC-like clone (3B11) from the same mouse (bottom panel; original magnification ×200). D, Comparison of cytology between the pCSCs, 3B11, and HSCs: HSC-enriched CD34^−^Lin^−^ and CD34^+^Lin^−^ cells were sorted by FACSorter from the BM of B6 mice (Wright-Giemsa staining; original magnification ×1000). DC-like cell line 3B11 was derived from the same mouse of the pCSCs. E, The karyotype of pCSCs: a representative of 2C4 clone that exhibits a pseudodiploid karyotype with multiple chromosomal translocations identical to the 3B5C and 3B6C clones (not shown). Left panel: karyotype of the 2C4 clone; right panel: an example of Robertsonian translocations in the 3B11 clone.

Although cancer stem-like cells can be detected in the existing tumor cell lines [17], no clonal CSC lines have been established. We previously reported that we cloned DC-like cell lines from the spleen of a leukemic mouse [14]. Here, we found that 3 of the 25 clones (2C4, 3B5C and 3B6C) failed to express hematopoietic pan-marker CD45 and lineage (Lin) markers CD3ε, CD4, CD8, B220, Ter-119, CD11b and Gr-1 (Fig. 1a), while the remaining clones, such as 3B11, exhibited monocytic and B cell phenotypes, expressing CD45, CD11b, B220, and CD19 (Fig. 1b). Further analysis of the stem cell-related markers revealed a unique phenotype– CD34^−^, CD38^low^, c-Kit^−^ (K^−^), Sca-1^−^ (S^−^), CD90^−^, Ly6C^−^ and CD44^high^ (Fig. 1a)– somewhat distinct from normal hematopoietic stem/progenitor cells reported previously [18] and the 3B11 clone shown in Fig. 1b. Cytological analysis demonstrated that all the clones exhibited blast-like cell morphology with large numbers of cytoplasmic vacuoles or granules. This morphology was distinct from the DC clones, such as the 3B11 clone of the same mouse [14], but comparable to the normal BM-derived CD34^+^Lin^−^ and CD34^−^Lin^−^ blast cells (Fig. 1c & d). Overall, these clones exhibit a stem-like cell phenotype CD45^−^c-kit^−^Sca-1^−^Lin^−^CD44^high^ (CD45KSL^−^CD44^high^). Cytogenetic analysis revealed that all the three clones carried an identical pseudodiploid karyotype with multiple chromosomal translocations. There are 40 chromosomes, and a sex chromosome (X or Y) is missing (Fig. 1e). In contrast, DC-like cells 3B11 from the same mouse are hyperdiploid with 46∼54 chromosomes and marked by karyotypic instability and several abnormal metacentric and submetacentric chromosomes, frequently resulting from Robertsonian translocations (Fig. 1e). These metacentric and submetacentric chromosomes varied from cell to cell. None of the cells were identical because of the marked karyotypic instability. Overall, the pCSCs exhibited a phenotype of CD45^−^c-kit^−^Sca-1^−^Lin^−^CD44^high^ or CD45KSL^−^CD44^high^ with multiple cytogenetic alterations. They are phenotypically and cytogenetically distinct from the differentiated cancer cells in the same host.

### 2. pCSCs have incompletely abolished multipotency

To test whether the pCSCs have the activity of hematopoietic progenitors, we evaluated the multipotency of pCSCs using the colony-forming cell (CFC) assay [19]. About 30∼50% of the input cells had colony-forming activity (CFU) in the medium of Methocult GF M3434 (Figure S1a). Although we did not observe all types of CFUs that may be differentiated from normal HSCs, such as burst forming units-erythroid (BFU-E), CFU-M (macrophage), and CFU-G (granulocyte), three types of CFUs were identified from these clones, including CFU-E (erythroid), CFU-mix, and CFU-GM (Fig. S1b). Accordingly, early lineage differentiation markers hemoglobin Hbb-1 for erythrocytes, CD41 for megakaryocytes [20], and c-fms for granulocytes/macrophage [21] were either constitutively expressed (i.e. CD41) or up-regulated (i.e. Hbb1 and c-fms), as demonstrated by RT-PCR (Fig. S1c). Conversely, erythropoietin receptor (EpoR) [22], von Willebrand factor (vWF) [23], and granulocyte colony stimulating factor receptor (G-CSFR), which are differentiation markers for the late stages of, respectively, erythrocytes, megakaryocytes and granulocytes/monocytes/macrophages, were undetectable in all the clones of pCSCs (Fig. S1c). These results suggest that the normal development program of the pCSCs was dramatically impaired, but not completely abolished.

To confirm our results, we cultured the pCSCs in suspension medium in the presence of myeloid or lymphoid lineage-specific cytokines. In the presence of G-CSF [24], all the pCSCs clones proliferated, and the cumulative cell number increased until the differentiated myeloid cells were morphologically identified (Fig. S2b; between d9 and d13). Then, the cell numbers progressively decreased, and almost all the cells died within 3 wks of culture (Fig. S2a, b, and data not shown). Because the cultures were split at the log phase in an attempt to prevent cell death caused by overgrowth, the cell death appeared to be associated with their inability to complete the differentiating program, even though some of the cells had acquired the ability to phagocytose apoptotic cells (Fig. S2b, d17). In addition, GM-CSF also induced cell death. For example, 2C4 cells died at day 6∼7 of culture while in the presence of recombinant murine GM-CSF (Fig. S2c). This suggests that it was the GM-CSF-mediated differentiating program, not the G-CSF-mediated one, which led to their earlier death. Moreover, the pCSCs also have the potential to differentiate into lymphoid cells when co-cultured with IL-7 and/or IL-15. This was shown when lymphoid markers B220 and NK1.1 were up-regulated on the pCSCs, albeit the markers were expressed variably (Fig. S2d). However, they also failed to complete the differentiating program and underwent apoptosis within two wks of culture (Fig. S2e, d12). In contrast, 3B11 cells did not die when cultured in the presence of G-CSF (data not shown). Taken together, the results suggest that the pCSCs retain incomplete potency of differentiation toward various hematopoietic lineages.

### 3. pCSCs have long-term repopulating activity

**Figure 2:**
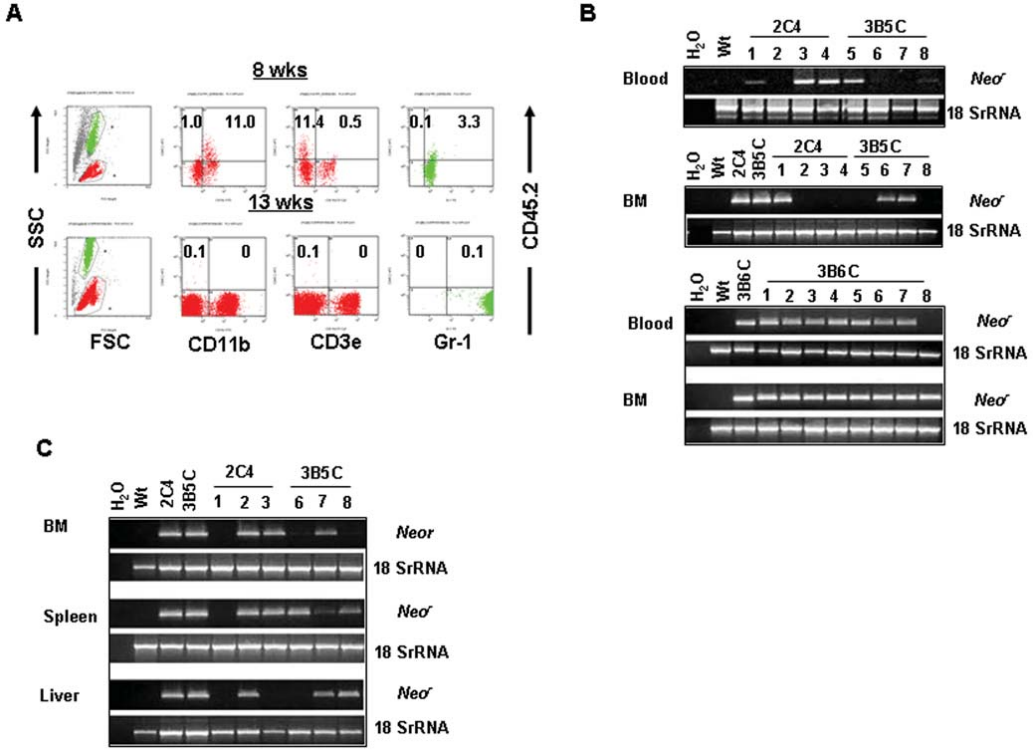
pCSCs have long-term repopulating activity. Congenic CD45.1 B6 mice were lethally irradiated and injected i.v. with 0.5∼1×10^6^ 2C4, 3B5C or 3B6C cells along with 2∼5×10^5^ recipient-type BM cells. Donor-specific CD45.2^+^ lymphoid (CD3ε^+^) and myeloid (CD11b^+^ or Gr-1^+^) cells were monitored by flow cytometric analysis of blood cells starting from 4 wks post transfer, once every two wks, until 18 wks (A). The mice were sacrificed 10 moths post transfer, and the blood and BM cells were collected for HANDS-Nested DNA PCR to identify donor-derived cells (B). To verify the self-renewal capability of the long-term repopulated donor cells, 1×10^6^ BM cells from the primary recipients were injected i.v. into the secondary recipients, which were sacrificed 10 wks post transfer. The pCSC-derived *neo^r^* gene in the BM, liver, and spleen was determined by HANDS-Nested DNA PCR (C). The data shown in B are from a recipient with transient expansion of pCSC-derived hematopoietic cells at 8 and 13 wks post transfer, and the data shown in C & D are from one of 3 experiments (5∼10 mice/group/expt).

The aborted *in vitro* hematopoietic differentiation may indicate how strict environmental cues influence pCSC differentiation and/or survival. To test this hypothesis, a competitive *in vivo* repopulating assay was performed [19]. Lethally irradiated CD45.1^+^ congenic B6 mice were injected i.v. with both 2C4, 3B5C or 3B6C cells and recipient-type bone marrow (BM) cells. The latter, BM cells were added to the injection to minimize recipient deaths due to lethal irradiation. Donor-derived CD45.2^+^ lymphoid (CD3ε^+^) and myeloid (CD11b^+^ or Gr-1^+^) cells in the peripheral blood were monitored by flow cytometry beginning ∼4 wks post-transplant. CD45.2^+^ donor cells were not significantly detected until ∼8 wks after transfer (Fig. 2a). About 0.5∼10% more CD45.2^+^CD11b^+^ and CD45.2^+^Gr-1^+^ cells were detected, depending on individual mice or experiments (5∼10 mice/expt) (Fig. 2a). Two weeks later, the pCSC-derived CD45.2^+^ cells decreased unexpectedly to less than 0.5% in the peripheral blood and were gone at ∼13 wks (Fig. 2a). These results suggest that, like our experiments performed *in vitro*, the pCSCs also have multipotent but incomplete differentiation *in vivo,* eventually leading to cell apoptosis. The incomplete differentiation may explain the low myeloid engraftment, as well as the absence of lymphoid engraftment. We define the process as differentiation-induced cell death (DICD), probably a protective mechanism that prevents pCSCs from progressing to malignant cells.

In addition to DICD, the transient *in vivo* expansion of pCSC-derived hematopoietic cells in the recipients (Fig. 2a) might be associated with the pCSC that lacks long-term repopulating activity. To test this possibility, a neomycin-resistant (*neo^r^*) gene was used as an indicator of long-term repopulating activity in the competitive repopulating assay, as described above, because the pCSCs were derived from a p53^−/−^Stat-1^−/−^ mouse, which carried genome-integrated *neo^r^* gene [12], [13]. The *neo^r^* gene was detected in the blood or BM cells of recipients even 10 months after transplantation by HANDS-Nested DNA-PCR, a technique that combines HANDS (Homo-Tag Assisted No-Dimer System) PCR [25], [26] with Nested PCR [27] and is used to ensure the specificity and sensitivity of detection. The *neo^r^* was detected in all of the recipients, though not in all the tissues examined (Fig. 2b). The repopulating activity was transferable because the *neo^r^* was detected in various organs of secondary recipients that received BM cells from the primary recipients (Fig. 2c). Surprisingly, while the *neo^r^* gene was undetectable in the BM cells of some primary recipients (Fig. 2b), it was readily detected in the secondary recipients receiving these BM cells (Fig. 2c). Thus, the lack of detectable *neo^r^* in the BM of primary recipients does not show that pCSCs are unable to repopulate the BM of these recipients; rather, it reflects the presence of either rare, quiescent pCSCs that repopulated the BM of primary recipients or an extremely low frequency of pCSCs resulting from expansion followed by apoptosis, as shown in Fig. 2a. These results suggest that pCSCs have the capacity of long-term repopulation.

### 4. pCSCs can differentiate into various types of nonmalignant cells

The lethally irradiated mice receiving both pCSCs and BM cells survived tumor-free for up to 10 months, except for 10∼20% of the mice, which died within 10 d after injection, probably due to an effect of the irradiation. In addition to the BM and blood, *neo^r^* was also detected in the liver, kidney, small intestine, heart and/or lungs of both primary (Fig. S3) and secondary recipients (Fig. 2b), indicating that the pCSCs either distributed in various organs in a quiescent status or differentiated into tissue-specific cells in these organs or tissues.

**Figure 3:**
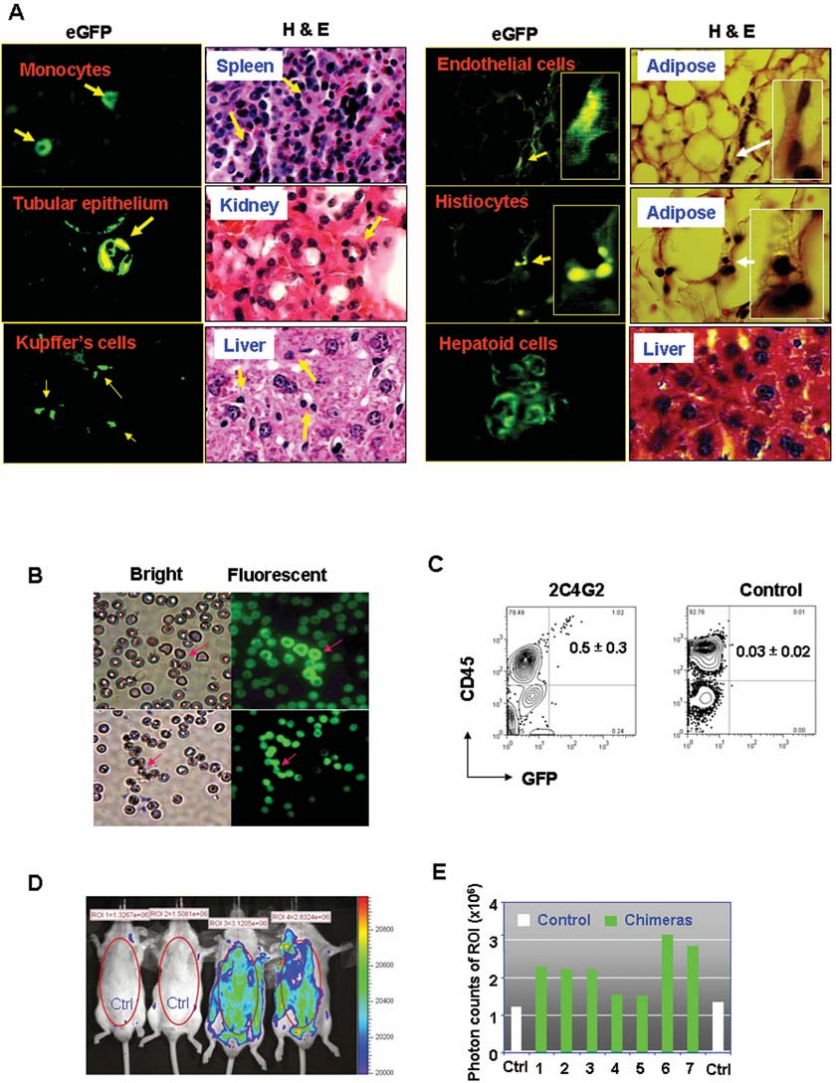
pCSCs can differentiate into various type of tissue cells. A, Differentiation of pCSCs into hematopoietic and non-hematopoietic cells: The lethally irradiated CD45.1 congenic B6 mice were injected i.v. with 1×10^6^ 2C4 (n = 5) or eGFP^+^ 2C4G2 cells (n = 10), along with 5×10^5^ recipient-type BM cells, as described above. The mice were sacrificed 5 months post transfer. Various organs, including the liver, kidney, spleen and adipose tissues were harvested, fixed in 10% formaldehyde of PBS, prepared for H & E. staining, and examined under fluorescent microscope. At least three discontinuous sections (100 µm/step) were examined for each organ to ensure that eGFP^+^ cells were identified under the fluorescent microscope. The morphology of eGFP^+^ cells was determined under the bright field of the fluorescent microscope (original magnification ×1000). B–E, Development of pCSCs in blastocyst chimeric mice: E3.5 dpc of FVB mice were injected with 2C4G2 (8∼10 cells per blastocyst) and transferred to pseudopregnant surrogate mothers. The progeny were delivered and grew to adult without any complication. The data shown are from one of two experiments in which 8 progeny (male: n = 6; female: n = 2) were obtained. One male mouse died of fighting at 3 months of age. B, eGFP^+^ RBCs in 7/8 of the chimeric mice: The data represent the air-dried blood smears from two mice, at the age of 2 months, examined under, respectively, the bright and fluorescent fields of a fluorescent microscope (Nike, E400, Japan). C, pCSC-derived eGFP^+^CD45^+^ cells: peripheral blood was harvested from the chimeric mice at age 2 months (n = 6; other two pregnant mice were not examined) or control FVB mice (n = 10), stained with PE-conjugated mAb to CD45, and analyzed by flow cytometry. D & E, Living image of the chimeric mice: A representative living image of the chimeric mice at 4 months of age is shown in D, demonstrated by IVIS imaging systems incorporated with Living Imaging® software (Xenogen Inc.); and the eGFP-derived photon counts in the region of interest (ROI) of 7 mice are shown in E. Normal FVB mice were used as control for living imaging.

To test these two possibilities, we stably transduced 2C4 cells with lentiviruses, which carried an enhanced green fluorescent protein (eGFP) gene, to track the fate of pCSCs in recipients. We obtained several clones, such as clone 2C4G2 (Fig. S4) that expressed a high level of eGFP. The 2C4G2 and parent 2C4 cells were intravenously transplanted, respectively, into lethally irradiated, BM-reconstituted CD45.1 congenic mice, which should provide stem cells with a vigorously regenerative environmental cue. The donor-derived eGFP^+^ cells, albeit lower in frequency, were readily detected in various organs, such as the spleen, liver, kidney, small intestine, or adipose tissues, of all the mice that had received 2C4G2, but not 2C4 cells, for 5 months (Fig. 3a and data not shown). Some eGFP^+^ cells exhibited the morphology of tissue origin, including endothelial cells, tubular epithelial cells, Kupffer's cells, histiocytes, macrophages/monocytes, and hepatoid cells (Fig. 3a). In the liver, eGFP^+^ Kupffer's cells and hepatoid cells were usually found in the regenerative areas (Fig. 3a). Interestingly, none of the observed eGFP^+^ cells exhibited significant dysplastic changes (Fig. 3a). The results suggest that while the pCSCs were generally unable to completely differentiate into mature hematopoietic cells *in vitro* (Fig. S1 & S2); some of them may differentiate into tissue-specific cells in a regenerative environment. Interestingly, none of the recipients developed tumors under the conditions.

To confirm the importance of environmental cues for the differentiation of pCSCs, we investigated their fates during embryo development using a blastocyst complementary assay, in which abnormal genetic programs may be corrected [28]. 2C4G2 or 2C4 cells were injected into 3.5 d old murine blastocysts (∼8 cells per blastocyst). The chimeric progeny were delivered from foster mothers and grew to healthy adults. As shown in Fig. 3b, eGFP^+^ red blood cells (RBCs) were detected in the peripheral blood of 88% (7/8) of the 2C4G2 chimeric mice, although their morphology was subnormal compared to the host RBCs, and the eGFP protein was variably expressed. The number of eGFP^+^ RBC was <50 in each slide of blood smears. About 0.5% of the gated white blood cells (WBCs) expressed both eGFP and CD45, as revealed by flow cytometry (Fig. 3c). Consistently eGFP signals were also detected in all 2C4G2 chimeric mice by IVIS™ imaging systems (Xenogen Inc.) (Fig. 3d & e). The results suggest that the pCSCs bypassed the checkpoint of differentiation-induced cell death, developed “normally” in the blastocysts, and differentiated into RBCs and WBCs in the chimera mice despite of low frequency. Importantly, the chimeric mice did not develop tumors within 20 months of follow up. The results indicate that the embryonic (juvenile) environment can support “benign” development of pCSCs, probably through correcting an altered oncogenic genetic program.

Taken together, we propose that pCSCs have the potential to differentiate into various types of nonmalignant tissue cells within appropriate environmental cues.

### 5. pCSCs develop into cancers in immunodeficient but not in immunocompetent mice

While pCSCs were detected in the lethally irradiated, BM reconstituted mice (Fig. 2a–c & 3a) and blastocyst chimeras (Fig. 3b–e), they developed into neither leukemic nor solid tumors after i.v. injection, despite that they were immortalized *in vitro*. This may be due to the immune surveillance system [29], which eliminated the pCSCs when they were progressing to CSCs or cancer cells, or possibly due to the route of cell delivery. The route of injection can dictate the environmental conditions, which effects the developmental fate of pCSCs.

**Figure 4:**
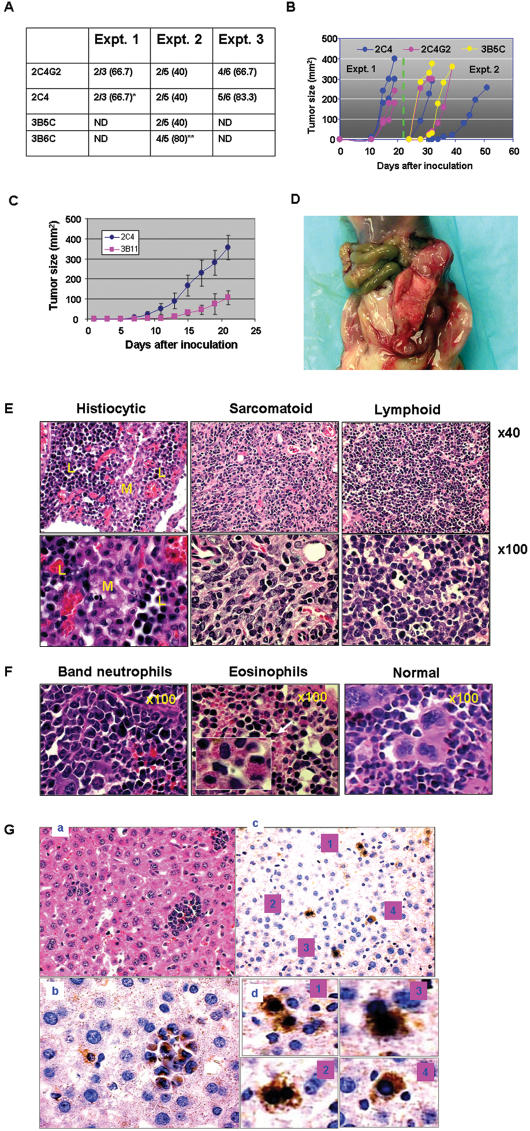
pCSCs developed into various type of tumors in immunodeficient mice. A, Tumor incidence from 3 experiments. Equal numbers of sex matched SCID mice were injected s.c. or i.p. with 5×10^6^ pCSCs. No significant difference in incidence was observed between s.c. and i.p. injected mice. As a control, C57BL/6 mice injected s.c. (n = 10) or i.p. (n = 10) with 2C4 cells did not develop tumors within 5 months of observation (data not shown) “*” indicates that a mouse developed ascites, “**” indicates that the 3B6C cells infiltrated in the liver and spleen (see E). B, Kinetics of tumor growth: the data shown are from experiments 1 & 2 in A. Each color in B corresponds to a specific cell line. C, Comparison of tumorigenesis between pCSCs (2C4) and differentiated cancer cells (3B11); (n = 10/group, each group includes 5 males and 5 females). D, A representative of gross tumors from a mouse injected i.p. with 3B5C clone. E, A histological representative of pCSC-derived tumors from the mice injected i.p. with 2C4 or 3B5C clones. F, A histological representative from the spleen of mice injected i.p. or s.c. with 3B6C clone. Note that megakaryocytes in the spleen of normal SCID mice were replaced by atypical neutrophils or eosinophils. G, Benign differentiation of pCSCs in the liver with metastatic cancers: (a) H & E staining of a liver section with metastatic cancers from a mouse injected i.p. with 2C4 cells (original magnification: ×200). (b) Immunohistochemical staining of the liver section from the same mouse with antibody to neomycin, showing neomycin^+^ cancer cells (original magnification: ×400). (c) Immunohistochemical staining of pCSC-derived hepatoid cells in the regenerative area of the liver sections from the same mouse (original magnification: ×200). (d) The enlarged micrographs of hepatoid cells demonstrated in (c).

To test this hypothesis, pCSCs were injected s.c., i.p. or i.v. into severe combined immune deficient (SCID) mice, BM reconstituted, or naive B6 mice; conditions which may provide different levels of immune surveillance (see discussion). The results demonstrated that both the surveillance system and the injection route had an impact on pCSC development in recipients; they developed into either solid or leukemic tumors in immunodeficient mice (Supplementary Table S1). 2C4, 2C4G2 and 3B5C cells, but not 3B6C cells, developed into solid tumors at the site of injection, regardless of i.p. or s.c. inoculation. The latency, incidence, and growth kinetics of tumors were also variable with the experiments (Fig. 4a &b). For example, the tumors were palpable, respectively, at days 10 and 21 post inoculation in the expt 1 and expt 2 (Fig. 4b). In most of the experiments, once tumors were palpable, the tumor growth curves rose steeply, and the mice had to be sacrificed within one wk (Fig. 4b). Consistent with the properties of tumorigenic cancer cells [5], pCSC-derived tumors grew much faster than 3B11 cell-derived tumors (Fig. 4c).

Some large tumors showed gelatinous areas with sharply delineated regions of tan-pink and gray (Fig. 4d), suggesting that the composition of the tumors was heterogeneous. Consistent with their gross appearance, the tumors were composed of various types of cancer cells, such as lymphoid, sarcomatoid (spindle) and histiocytic cancer cells (Fig. 4e).

Although 3B6C cells did not develop into a solid tumor in situ, they infiltrated the spleens of about 80% of recipient mice, resembling chronic leukemic alterations (Fig. 4a & f). Large numbers of atypical neutrophils or eosinophils were observed in the spleen (Fig. 4f), suggesting that 3B6C cells were distinct from 2C4 and 3B5C clones with regard to the resultant tumor type, although the karyotype between them were identical (Fig. 1e and not shown). The results indicate that pCSCs from a single clone can differentiate into various types of cancer cells.

In addition, metastatic cancers were detected in the spleen, liver, prostate, pancreas, and brain, but not the lungs (Fig. S5 and not shown). Some of them demonstrated spindle/oval cell morphology (Fig. S5). Interestingly, benign differentiation of pCSCs in the liver with metastatic cancer was also observed in the regenerative area of liver parenchyma, which was revealed by the scattered neomycin-positive hepatoid cells (Fig. 4g), suggesting that the benign and malignant differentiations of pCSCs are delicately regulated by microenvironments.

The i.v. injected pCSCs, interestingly, did not develop into tumors in the solid organs of SCID mice, even at 5 months post injection (Supplementary Table S1). However, chronic leukemic alterations were observed in the spleens of all the mice injected with 2C4, 3B5C or 3B6C clones (Fig. S6b). Fewer pCSC-like cells were detected in the peripheral blood (Fig. S6c). Moreover, all the organs examined, including the liver, kidney, lungs, small intestine and pancreas were histologically normal (data not shown). In contrast, no leukemic alterations were observed in the spleens of either the naive or BM-reconstituted immunocompetent B6 mice, which were injected i.v. with 2C4, 3B5C or 3B6C cells (Fig. S6d; Supplementary Table S1). Taken together, the results suggest several important points for the tumorigenesis of the pCSCs. First, the pCSCs require an appropriate environmental cue, such as tissue extracellular matrix [30], to acquire tumorigenicity. This may explain why the pCSCs can develop into solid tumors only when injected s.c. or i.p., but not i.v. Second, the immune system may suppress pCSCs progressing to cancer cells because they did not develop into tumors when injected into immunocompetent mice. Finally, the pCSCs may represent an early developmental stage of CSCs, namely precancerous stage; depending on the environmental cues. They may undergo either benign or malignant differentiation or remain quiescent.

### 6. Phenotypic and genetic alterations when pCSCs progress to cancer

**Figure 5:**
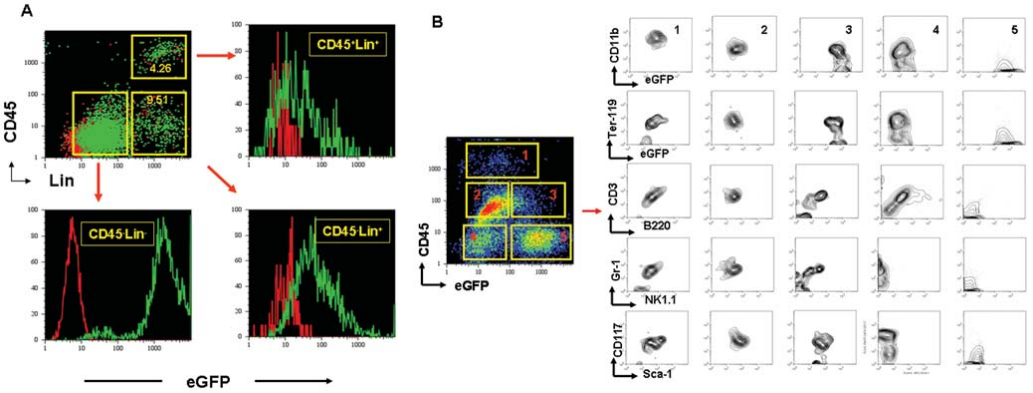
Phenotypic alterations of pCSCs progressing to cancer. Single tumor cells were either prepared and freshly stained with mAb to CD45 and a mixture of lineage-specific mAbs to CD3, CD11b, Ter-119, Gr-1 and B220 (A) or cultured for 2 d and stained with mAb to CD45 in combination with mAbs to lineage markers or to c-kit and Sca-1 as indicated (B). The samples were analyzed by three- (A) or four-color flow cytometry (B). The green and red dot plots or histograms represent, respectively, the tumor cells derived from eGFP^+^ 2C4G2 (green) and eGFP^−^ 2C4 (red) cells (A). Five populations (p1∼5) of tumor cells are identified based on the level of CD45 and eGFP expression (B).

If the pCSCs are at an early stage of cancer development, we should observe the phenotypic and genetic changes as they become cancerous. To verify the hypothesis, we analyzed 2C4 and 2C4G2 cell-derived lymphomas. As shown in Fig. 5A, CD45^+^Lin^+^ and CD45^−^Lin^+^ tumor cells were detected in both tumors. These cells developed from the injected pCSCs because both populations expressed eGFP in the 2C4G2-, but not in the 2C4-derived tumors. Interestingly, eGFP was somewhat down-regulated when the pCSCs differentiated into Lin^+^ cells (Fig. 5A). Further analysis of the tumor cells revealed that after 2 days of culture, CD45^+^eGFP^low or +^ (p1, 2 & 3) and CD45^−^eGFP^low^ populations (p4) abnormally expressed all the lineage markers (CD3ε, CD11b, B220, Ter-119 and Gr-1) with variable levels. Other lineage markers, such as NK1.1, were also expressed when CD45 was up-regulated (Fig. 5b: p1, 2 & 3). Importantly, the up-regulation of CD45 and lineage markers was accompanied by the expression of c-kit (CD117) (Fig. 5b: p1∼4) and Sca-1 (Fig. 5b: p1∼3). The expression of CD117 and Sca-1 may signify the malignancy of the Lin^+^ cells, because CD117, a transmembrane tyrosine kinase receptor encoded by proto-oncogene c-kit, and Sca-1, a glycosylphosphatiylinositol-linked cell surface protein, have been identified as markers of cancer progression in various types of cancer [31]–[33]. Based on the results (Fig. 5a & b), we propose a road map for the pCSCs progressing to cancer cells: CD45^−^c-kit^−^Sca-1^−^Lin^−^→CD45^−^c-kit^+^Sca-1^−^Lin^+^→CD45^+^c-Kit^+^Sca-1^+^Lin^+^. Further characterization of the populations is ongoing in our laboratory.

Based on the genetic model of colorectal tumorigenesis [34], we assumed that the karyotype of pCSCs might be altered as they progressed to cancer. To test the hypothesis, we analyzed the karyotypes of pCSC-derived tumors. Although the karyotypes of the tumor cells were the same as the parental pCSCs (Fig. 1e), we found a few changes. Compared to the parental line karyotype, for example, one 2C4-derived tumor cell line exhibited a deleted 10 and an add [14] in 90.6% (29/32) of the cells, while the other 3 cells had the dup [14] and a third copy of the del [15] found in the pCSCs, thus showing karyotypic evolution in both clones of this line compared with the parental line. Thus, genetic alteration was dynamic when pCSCs were progressing to cancer.

### 7. The ectopically expressed *piwil2* gene regulates pCSC expansion *in vitro*

**Figure 6:**
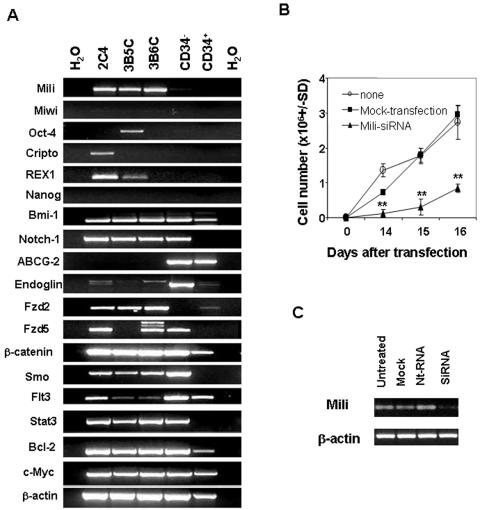
Exclusive expression of *piwil2* and embryonic stem cell-related genes in pCSCs. A, Exclusive expression of *mili* (*piwil2*) gene in pCSCs: Total RNA was isolated randomly from 2C4, 3B5C and 3B6C cell cultures at various times or from the CD34^+^Lin^−^ and CD34^−^Lin^−^ BM cells of B6 mice, which were purified by FACS Aria in 3 separate experiments, and was subject to RT-PCR analysis for embryonic, germ-line, and adult stem cell-related stemness genes and oncogenic genes. The data represent at least 3 experiments. B, Inhibition of pCSC expansion *in vitro* by mili-specific siRNA: 2C4 cells (100 cells/well) were either transfected or not by mili-specific siRNA (100 nMol) or mock-transfected in triplicate in 24-well plates. The cells were counted at indicated times. The data represent 5 experiments. **, p<0.01 as compared to the mock- or non-transfected groups. C, Knockdown of *mili* mRNA by mili-specific siRNA: 2C4 cells (1×10^6^/well) were transfected by mili-siRNA or scramble nucleotide (nt) RNA, and harvested 48 hrs post transfection. The expression of *mili* mRNA was revealed by RT-PCR. The data represent 3 experiments.

Since the pCSCs exhibited the properties of stem cells, as well as the potency of tumorigenesis, we examined the expression of embryonic and adult stemness-related genes in these cells: *mili (*a mouse homologous of *piwil2)* [15], *Bmi-1* [35], *Notch-1* [36], *Endoglin* [37], *ABCG-2* [38], *POUF1/Oct-4* [39], *Nanog* [39], *TDGF1/Cripto* [40], *Zfp42/REX1* [41], *Fzd2* [42], *Fzd5* [43], *β-catenin* [44], *Smo* [45], *c-Myc* [46], *Flt3* [47], *Bcl-2* [48] and *Stat-3* [49]. Some of these genes are also associated with tumor development [50]. BM-derived CD34^+^Lin^−^ and CD34^−^Lin^−^ cells, which were enriched with hematopoietic stem cells (HSCs), were used for comparison. As shown in Fig. 6a, both embryonic and adult stem cell-related genes were detected in the pCSCs, except for *Nanog* and *ABCG-2*. Adult stem cell- and tumorigenesis-related genes, such as *Bmi-1, Notch-1, Fzd2, Fzd5, Flt3, Smo, β-catenin, Stat-3, and Bcl-2* were detected in both pCSCs and normal stem cells (NSCs). Interestingly, embryonic stem cell-related genes, including *Pouf1/Otc4, TDGF1, Zfp42/REX1* and *Mili (piwil2)*, whose homologue has a conserved function in stem cell division [16], were exclusively expressed in pCSCs. Among them, only *mili* was stably expressed in all the clones of pCSCs; in contrast, *miwi* [15], a member of mouse PIWI/AGO gene family, was not detectable in these pCSCs (Fig. 6a), although its human homologue *hiwi* was detected in human CD34^+^ HSCs [51]. The unique pattern of *mili* and *miwi* mRNA in pCSCs does not seem to be associated with the deficiency of p53 and Stat-1, because we could not detect either gene in the p53-null embryonic fibroblasts or the Stat-1-null hematopoietic cells (data not shown). The results suggest that *mili* may play an important role in pCSC development. Thus, we further examined the effect of *mili* on pCSC expansion *in vitro*. We knocked down the *mili* gene in 2C4 cells using *mili*-specific siRNA, resulting in a significant decrease of 2C4 cell expansion (Fig. 6b & c). The results suggest that *mili* promotes pCSC proliferation, which is consistent with the recent observations for the *mili*-overexpressing NIH3T3 cell line [52].

**Figure 7:**
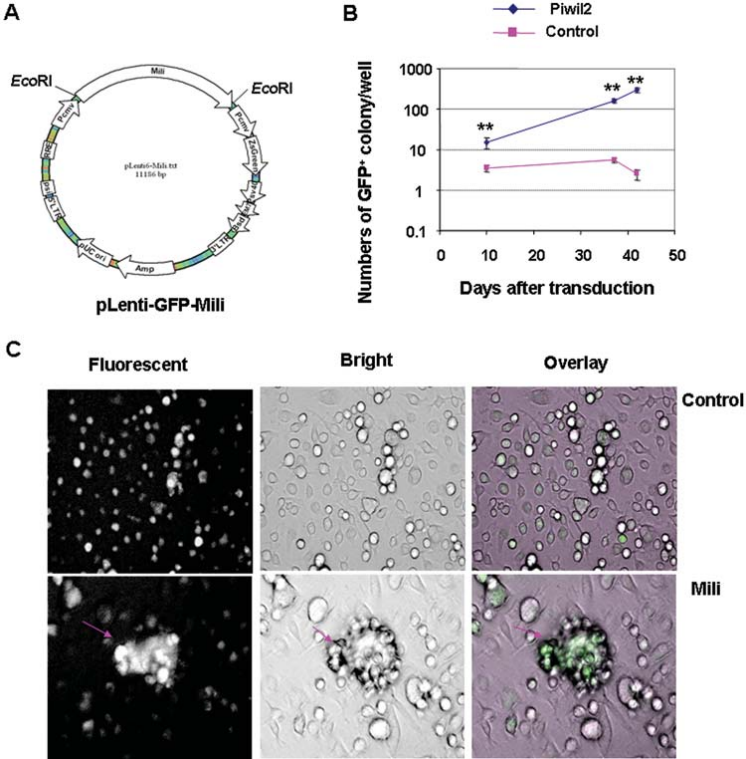
Ectopic expression of *mili* in BM cells promotes cell proliferation. A. Schematic construct of Lenti-GFP-Mili viral vector. B, The effect of ectopic expression of *mili* on marrow cell proliferation: BM cells from B6 mice were transduced with Lenti-GFP-Mili or Lenti-GFP viruses in 24-well plates (n = 4 well/group), as described in Methods. The number of GFP^+^ colonies were counted at indicated times. **, p<0.01, compared to Lenti-GFP group. C, Representative eGFP^+^ EB-like colonies at day 10 post transduction (arrow). The micrographs were taken under the inverted fluorescent microscope (Nikon, TE2000-U, Japan), using original magnification: ×200.

To confirm this idea, we transduced murine BM cells with Lenti-GFP-Mili or Lenti-GFP pseudoviruses (Fig. 7a) in the XLCM-conditioned medium [53]. After 10 days of transduction, a number of eGFP^+^ colonies were observed in the cultures of BM cells transduced with Lenti-GFP-Mili, whereas considerably fewer eGFP^+^ colonies were demonstrated in the control cultures, despite the comparable efficiency of transduction (Fig. 7b & c). Interestingly, some embryonic body (EB)-like colonies were observed in the cultures of *mili*-transduced BM cells (Fig. 7c). The results verify that ectopic expression of *mili* promoted cell proliferation. Thus, it is likely that ectopic expression of *mili* may contribute to the development of pCSCs. However, *mili* was not required for the self-renew of normal hematopoietic stem cells (HSCs), because the number and repopulation activity of the HSCs from the BM of *mili*-disrupted mice was not affected [15].

## Discussion

Though cancer can arise from a stem-like cell, namely CSC, little is known about the mechanisms for CSC development. Extensive investigations have revealed that human tumorigenesis is a complex, multistep process often requiring a concordant expression of several genes, including multiple genetic and epigenetic alterations in oncogenes, tumor-suppressor genes, cell-cycle regulators, cell adhesion molecules and DNA repair genes, genetic instability, as well as telomerase activation [34], [54]. Since stem cells are long-lived cells, they are the likely subject of accumulating mutations that lead to their malignant transformation and tumor initiation [1], [10]. Thus, the cells that hierarchically transit from normal stem cells or committed tissue progenitor cells to CSCs are defined as precancerous stem cells (pCSCs), which may be uncommitted with incomplete multipotency. However, they seem to be predetermined, depending on environmental cues, in the formation of solid or leukemic tumors.

**Figure 8:**
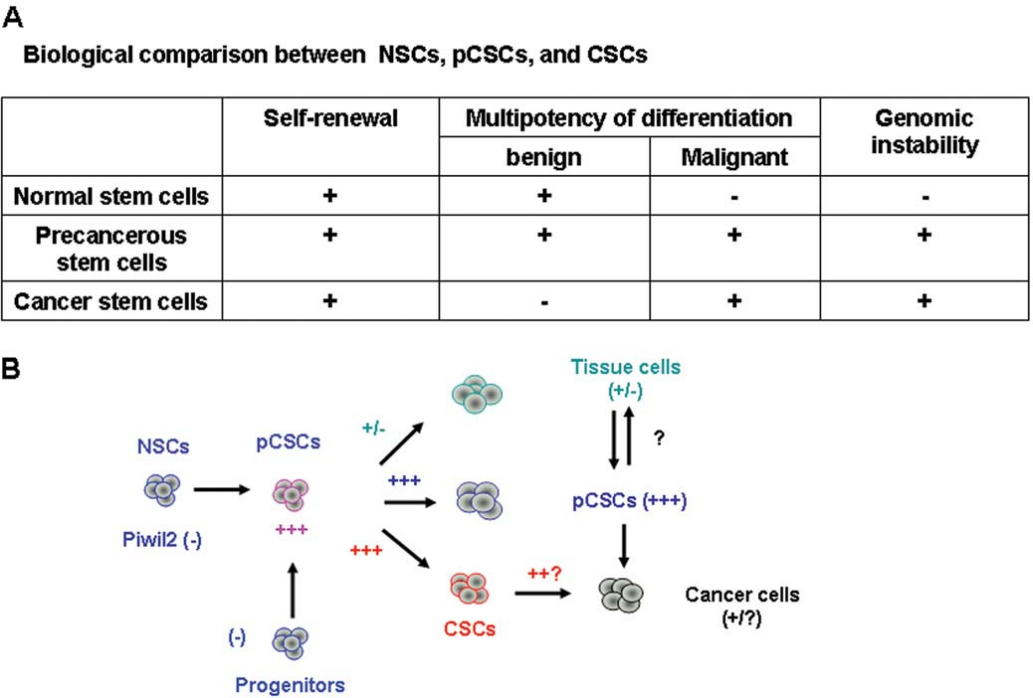
The developmental relationship between pCSC, CSC and cancer. A, Biological comparison between NSCs, pCSCs, and CSCs. B, Schematic model of pCSC development: *piwil2* might play an important role in pCSC development.

We experimentally defined several stem-like cell clones as pCSCs because they have the potential for both benign and malignant differentiation, seemingly reflecting an early stage of CSC development (Fig. 8). The pCSCs have the characteristics of a stem-like cell: self-renewal and multipotency of differentiation, albeit incomplete. The property of benign differentiation can distinguish them from CSCs, whereas the property of malignant differentiation can distinguish them from normal stem cells (NSCs) (Fig. 8a). In other words, pCSCs may represent an intermediate cell between normal and cancer stem cells if a cancer arises from a stem cell, or an intermediate cell between committed tissue cells and CSCs. The latter is likely should cancer arise from a committed progenitor cell, which has acquired stem-like cell properties. Thus, pCSCs is a paradoxical entity between normal and cancer stem cells. On the one hand, they appear to be uncommitted stem cells with incomplete multipotency; on the other hand, they seem to be pre-determined, depending on environmental cues, in the formation of solid or leukemic tumors. This is not surprising because the contradictorial properties of pCSCs reflect an important characteristic: their potential for benign and malignant differentiation.

However, the biological definition for pCSCs remains open, especially for its demarcation from CSCs [3]. Currently, human CSCs are experimentally defined based on their capacity to reconstitute cancer in immunodeficient mice [4], [5], [7]. It is not clear whether these CSCs could reconstitute cancers in the immunocompetent hosts. It is possible that these cells might be at the early stage of CSC development. At present, the hypothesis, however, is impossible to be tested in immunocompetent animals with human pCSCs. Animal cancer models can precisely define the stages of CSC development. It is commonly understood that some rodent cancer cell lines cannot reconstitute cancers when transplanted into immunocompetent hosts. These lines might have the properties of pCSCs, as we have described in this study.

In fact, the existence of pCSCs has been implicated in human LSCs. In the human acute myelogenous leukemia (AML), the frequency of LSCs is extremely low and is approximately 0.1∼1 per million AML blasts [4]. By tracking individual human LSCs in NOD/SCID mice serially transplanted with AML cells, LSCs were found not to be functionally homogeneous but, like the normal HSC compartment, comprise distinct hierarchically arranged LSC classes [55]. In this experimental model, two important features for LSCs/CSCs were revealed: 1) some LSCs are either quiescent or divide rarely and undergo self-renewal rather than commitment after cell division; and 2) normal developmental processes are not completely abolished during leukemogenesis [55], This probably reflect a precancerous stage of LSC/CSC development [4], [55], although it is not clear whether these cells, like pCSCs defined herein, have the potential for benign differentiation. In non-hematopoietic tumors, pCSCs may be responsible for the reversible precancerous lesions, such as metaplasia and dysplasia, in tumor pathology [10], although the notion remains to be substantiated.

The fate of pCSCs is determined by environmental cues. Our studies indicate that pCSCs can differentiate into benign cells in immunocompetent (IC) mice; or progress to cancer in SCID mice. This does not mean that pCSCs do not have the potential to progress to cancer in immunocompetent mice, or the potential to differentiate into benign cells in the SCID mice. The notion is supported by the fact that the pCSC-derived benign hepatoid cells were detected in the same liver of SCID mice with metastatic cancer. In the immunocompetent mice, the pCSC-derived malignant cells may be eliminated by the mechanisms of immunoediting. The benignly differentiated cells may not necessarily be “normal”, because they may still be genetically defective as their parent cells. However, these cells may be controllable in cell cycling, and thus are not malignant. In addition, these genetically defective cells may be susceptible to DICD or abortive differentiation, and only a few of them are alive and detectable. This scenario may explain the low engraftment of benign cells in the immunocompetent mice and blastocyst chimera mice. Thus, the benign differentiation of pCSCs signifies that the progeny of pCSCs may be eliminated by DICD and immunoediting, or sustained silent in an appropriate cue. In contrast, the malignant differentiation implicates the enhanced, uncontrollable cell survival and proliferation as well as resistance to DICD. This may be true because we have found that CSCs are more resistant to DICD than pCSCs upon stimulation by differentiating factors (our unpublished data). The notion is also supported in part by the fact that vaccination with pCSCs led to the resistance of hosts to the challenge by tumorigenic cancer cells (unpublished observation). The undifferentiated pCSCs may be restricted in expansion *in vivo* due to their clonal property, similarly to human LSC clones [4]. The low engraftment of a single clone also suggests a mechanism underlying the maintenance of homeostasis of pCSCs, namely programmed silencing, which is warranted for further investigation.

The pCSC-derived cells appear to be tissue-specific because each eGFP-expressing cell overlapped morphologically with the given tissue cells revealed by histology. Thus, we proposed that the pCSCs, like other BM-derived CSCs [6], have the potential of transdifferentiation, although we could not exclude the possibility of cell fusion between pCSCs and relevant tissue cells [56]. The benign transdifferentiation or the putative cell fusion probably provides one of the mechanisms preventing pCSC progressing to malignant cells.

The discovery of the potential of pCSCs for both benign and malignant differentiation helps explain the complex process of cancer development. Our studies suggest that the fate of developing pCSCs is determined by the status of the host immune system and the environmental cues (the site of cell colonization or route of inoculation). The pCSCs appear to be scrutinized by the mechanism of tumor immune surveillance [29] because the pCSC clones 2C4, 3B5C and 3B6C, which have an identical phenotype and karyotype, had different fates in three animal models with different levels of immune surveillance (Supplementary Table S1). They developed into neither solid nor leukemic tumors in native immunocompetent mice when injected s.c., i.p., or i.v. However, they developed into tumors (2C4 and 3B5C) or leukemia (3B6C) in the NK cell-sufficient but T and B cell-deficient SCID mice with a variable latency of tumor initiation and tumor incidence in separate experiments. Interestingly, while all the pCSC clones did not develop into chronic leukemia when injected i.v. into the lethally irradiated, BM-reconstituted mice with defective but recovering immune systems, they differentiated into non-malignant cells in the regenerative area of tissues. However, the pCSC-derived non-malignant cells might be able to re-dedifferentiate into pCSCs or CSCs in tumorigenic environments, leading to tumorigenesis (Fig. 8b). In addition, the pCSCs retained the capability of self-renewal, as evidenced by their transferable long-term repopulating activity. The long-term repopulation of pCSCs or pCSC-derived cells in various organs of the BM-reconstituted mice suggests that the immune system may not directly eliminate the quiescent pCSCs; rather, it may recognize pCSCs either undergoing DICD or developing to CSCs and eliminate them quickly. As a consequence, the quiescent pCSCs can be maintained in a limited clonal size. Thus, the fate of pCSCs is associated with the levels of tumor immune editing in the host [29]. Further analysis of pCSCs destiny, by regulating the level of immune editing, will help identify the mechanisms underlying the development and control of pCSC or CSCs.

In addition to tumor immune surveillance, pCSCs may also be checked by differentiation-induced cell death (DICD). Transient detection of pCSC-derived hematopoietic cells in the peripheral blood of the BM-reconstituted recipients suggests that the differentiating cells are short-lived and may spontaneously undergo apoptosis. To support this, the pCSCs underwent apoptosis when their incompletely abolished hematopoietic differentiation programs were activated by lineage-specific cytokines, such as G-CSF, GM-CSF, IL-7 and IL-15. Thus, DICD may prevent pCSCs from progressing to CSCs. Taken together, the fate of pCSCs is highly diversified and includes, depending on environmental cues, programmed silence, DICD, benign incomplete differentiation, elimination by immunoediting, and progression to cancer.

Because cancer can be caused by hierarchically genetic and epigenetic alterations [34], [54], it is difficult to define a common phenotype or genetic markers of CSCs with regard to their stemness and tissue origin [3]. In support of this notion, we demonstrated that the progression of pCSCs to cancer was accompanied by the up-regulation of c-kit and Sca-1, as well as by stable lineage markers, and increased genetic alterations. It would be difficult to phenotypically distinguish pCSCs from CSCs if not functionally. Further dissecting the developmental stages based on these markers and genetic alterations may lead to phenotypically distinguishing pCSCs from CSCs.

Genetically, it has also been difficult to define a gene marker for CSCs because the oncogenic genes expressed in CSCs are overlapped with those expressed in normal adult stem cells, such as such as *Bmi-1* [35]. We found, however, that pCSCs exclusively expressed embryonic stem cell (ESC)-related stemness genes, including *POUF1/Oct-4* [39], *TDGF1/Cripto* [40], and *Zfp42/REX1* [41], though the significance is not clear. It is possible that these genes confer pCSCs the multipotency of differentiation or the capability of benign differentiation. Although these genes were ambiguously expressed in pCSCs, they were undetectable in a CSC line established in our laboratory (Li et al., unpublished), suggesting that they may be subverted at the early stage of CSC development. Moreover, *piwil2* (alias *mili* in mouse *or hili* in humans), a member of PIWI/AGO gene family [16], which is exclusively expressed in testis and essential for stem cell self-renewal in *drosophila* [16], gametogenesis [15], and small RNA-mediated gene silencing [57], was stably expressed in pCSCs. The *piwil2* was also detected in various tumor cell lines of human and animals with variable levels [unpublished observation & [52]], probably related to the number of pCSCs and/or CSCs in each line. It is likely that ectopic expression of *piwil2* may contribute to the development of pCSCs and CSCs (Fig. 8b) because the knocking-down of *piwil2* mRNA led to the contained pCSC proliferation *in vitro*. Consistently, overexpression of *piwil2* in BM cells led to enhanced proliferation *in vitro*. These results are consistent with the recent observation from the *piwil2*-overexpressing NIH3T3 cell line [52], suggesting that the *piwil2* effects on pCSC development. However, the function of *piwil2* is complex than we imaged, for it may regulate large numbers of piwi-interacting RNA (piRNA) [58], [59]. Unexpectedly, the *piwil2* was not required for the self-renewal of HSCs, because the number and repopulation activity of hematopoietic stem cells from the BM of *mili*-disrupted mice was not affected [15]. Although the pCSC clones were derived from a p53^−/−^Stat-1^−/−^ mouse [14], p53 and Stat-1 has no direct effect on *piwil2* expression. Thus, the roles of ectopic expression of *piwil2* in pCSC development *in vivo* need further exploration.

It would be ideal to characterize the freshly isolated pCSCs from fresh tumor tissues at a single-cell level to avoid any possible results of due to cultural artifact. However, this is technically difficult because only exist rare numbers of pCSCs in tumors. Moreover, it is difficult to isolate a single pCSC based on its phenotype because pCSCs may be phenotypically heterogeneous due to their hierarchical or multiple genetic alterations. To prove the existence of pCSCs, we performed *ex vivo* limiting dilution at the beginning of our experiments [14] and obtained three pCSC clones with an identical karyotype. Thus, the data obtained from these pCSC clones appear to be at a single-cell level. It would be possible to further show the benign and malignant differentiation of these clonal pCSCs at a single-cell level. However, it was also difficult, and it may be related to the unique properties of differentiating pCSCs, such as their DICD, susceptibility to immunoediting, and programmed silencing. Even for CSCs, their existence has not been demonstrated at a single-cell level [4]–[9]. However, this does not exclude the existence of CSCs. Moreover, normal tissue stem cells, such as mammary epithelial stem cells, and their outgrowth at a single-cell level can be demonstrated in only one of several hundred transplants [60]. To further prove that pCSCs have the potential for benign and malignant differentiation, we performed blastocyst complementary experiments with only a few clonal pCSCs and verified the potency of benign differentiation of pCSCs in the blastocyst chimera. Moreover, the altered karyotype of pCSC-derived tumor cells, though nearly the same as parent cells, also suggests that pCSCs can progress to cancer.

Overall, we have experimentally identified and characterized the pCSCs, which may be important for understanding the mechanism underlying CSC development. Our findings provide the first evidence that single-cell pCSCs clones have the potential for both benign and malignant differentiation, depending on environmental cues. A broad therapeutic approach to the cure of various types of cancers may be achievable through rational targeting of pCSCs.

## Methods

### Experimental procedures

#### Mice, cell lines, and reagents

C57BL/c (B6) and SCID CB17 mice were used at the age of 8–12 wk. The mice were bred and maintained in the animal pathogen-free facility at The Ohio State University Medical Center. Cell lines 2C4, 3B5C and 3B6C were cloned from a mouse with dendritic cell-like leukemia, as previously described [14]. All mAbs and cytokines were purchased from, respectively, BD PharMingen and PeproTech, except where indicated.

#### Cell culture

The cell lines were maintained in R10F (RPMI 1640 plus 10% fetal calf serum supplemented with 5 mM glutamine, 50 µM 2-mecaptoethonal, 100 U/ml penicillin, and 100 µg/ml streptomycin) [14]. In some experiments, the cells were cultured in the presence or absence of cytokines, including the supernatant of G-CSF secreting U87MG cell culture [61], recombinant GM-CSF, IL-7 and IL-15. The cultures were split at the log phase of cell growth to prevent over population-induced cell death. The cytology was examined at various time points by Giemsa-staining of cytospin preparations, or directly monitored under a phase contrast microscope.

#### Purification of BM marrow stem cells

Lin^−^CD34^+^ and Lin^−^CD34^−^ BM cells were purified from B6 mice using MACS beads followed by FACS sorting. Briefly, BM cells were isolated and stained with a cocktail of biotinylated mAbs to lineage (Lin) markers CD3ε, CD11b, B220, Gr-1, and Ter-119, and then incubated with MACS beads coated with mAb to biotin (Miltenyi, Biotech Inc.). Lin^−^ cells were negatively selected, as instructed by the manufacturer (Miltenyi Biotech Inc.), stained with mAb to FITC-conjugated CD34 and PerCP-Cy5-conjugated streptavidin, and sorted for Lin^−^CD34^+^ and Lin^−^CD34^−^ populations using FACS Aria (BD Science). The purity of each population was >98%.

#### Generation of eGFP^+^ cell lines

To track the fate of pCSCs *in vivo*, 2C4 cells were transduced with pseudo-lentiviruses carrying an enhanced green fluorescent protein (eGFP) gene and a blasticidin-resistant gene. The eGFP^+^ cells were cloned by limiting dilution and maintained in R10F for more than 2 months. Flow cytometry was used to determine the clones that stably expressed eGFP. A clone, 2C4G2, which expressed the appropriate fluorescent intensity of eGFP was used for experiments, and parent 2C4 cells were used as a control.

#### Hematopoietic CFU assay

Mouse clonogenic hematopoietic progenitor assays were performed to assess the capacity of multilineage differentiation by pCSCs. pCSCs were plated into MethoCult GF M3434 (StemCell Technologies), and colonies (>50 cells) were scored after 12∼14 d incubation at 37°C and 5% CO_2_, as instructed by manufacturer.

#### *In vivo* competitive repopulating assay

pCSCs or eGFP^+^ pCSCs (0.5∼10×10^5^) were injected into the tail vein of lethally irradiated (900 rad) CD45.1 congenic B6 mice, along with or without 2∼5×10^5^ host-type BM cells. Donor-derived CD45.2 cells from peripheral blood were assessed by flow cytometric analysis for lymphoid (CD3^+^) and myeloid (CD11b^+^ and Gr-1^+^) cells every 2 wks, starting at 4 wks and ending 20 wks after transplant. Then the mice were maintained and sacrificed 5 or 10 months after transplant. Peripheral blood, BM, and various organs were harvested and examined, using HANDS-Nested DNA PCR, to determine if the *neo^r^* gene had integrated in the genome of donor cells.

#### Secondary repopulation assay

The BM cells (1×10^6^), isolated from primary recipient mice 10 months after transplant, were injected i.v. into lethally irradiated CD45.1 congenic B6 mice. The secondary recipients were sacrificed 5 moths after transplant. Donor-specific cells in the blood, BM, and liver were determined for the *neo^r^* gene using HANDS-Nested DNA PCR.

#### Tumorigenesis assay

SCID CB17 mice were injected i.p., s.c., or i.v. with 5×10^6^ pCSCs. Tumor incidence and size were monitored once every other day starting 1 wk after inoculation. The group was sacrificed when one of the mice developed a tumor more than 15∼20 mm in diameter. Tumors and various organs were harvested for histological analysis, immunochemical staining, and/or flow cytometric analysis.

#### Generation and characterization of blastocyst chimera mice

Four-week old FVB/N female mice (Taconic Farm) were superovulated by subcutaneous injection of 5 IU of Pregnant Mare's Serum (PMSG, NHPP) followed 46–48 h later with 5 IU of Human Chorionic Gonadotropin (hCG, Sigma). Females were subsequently mated with FVB/N stud male mice. Embryos were flushed from the uterine horn of the female mice (3.5 dpc) and then sacrificed. Blastocysts were collected and maintained in HEPES buffered CZB [62] during microinjection.

Microinjection was similar to injecting embryonic stem cells into blastocysts [62]. The Peizo-Micromanipulator [63] was used to inject 2C4G2 or 2C4 pCSCs. The injection pipette was prepared to the inner diameter 10∼15 µm with Narishige Microforge. Approximately 8∼10 cells were injected into fully expanded 3.5 dpc blastocysts. The injected blastocysts were incubated in CZB culturing medium [62] at 37°C, 5% CO2 incubator for 30 minutes and then surgically transferred to the oviduct of 0.5 dpc pseudopregnant ICR mice. Adult progeny was characterized for eGFP^+^ red blood cells (RBC) in peripheral blood under fluorescent microscope, CD45^+^eGFP^+^ white blood cells (WBC) by flow cytometry, and eGFP signals (photon counts) in the living body by IVIS imaging system 100 Series (Xenogen Inc.).

#### RT-PCR

Total RNA was extracted from cell lines or *de novo* isolated HSCs. The cDNA was generated by reverse transcription using Superscriptase II (Invitrogen, CA) and oligo (dT) in a 20 µl reaction containing 1 µg of total RNA, which was pretreated with RNase-free DNase I (Invitrogen, CA) to eliminate contaminating genomic DNA. Briefly, an aliquot of 0.5 µl cDNA was used in each 20 µl PCR reaction, using PCR Master Mix (Promega, Ca). The following conditions were used: an initial denaturation at 95°C for 5 min followed by denaturation at 94°C for 30 seconds, annealing at 65°C for 1 min, touchdown −1°C per cycle, and extension at 72°C for 1 min for a total of 10 cycles. Then the condition was fixed for 25 cycles of denaturation at 94°C for 30 seconds, annealing at 50°C for 1 min, and extension at 72°C for 1 min with a final extension at 72°C for 10 min. PCR products were analyzed by 1.5% agarose gel. The sequence of primers is listed in supplementary Table S2.

#### Genomic DNA isolation

Genomic DNAs of all tissues except blood were isolated following overnight digestion with 500 µl of DNA lysing buffer (100 mM NaCl, 10 mM Tris-HCl, 25 mM EDTA, 1%SDS, and 50 µg/ml proteinase K) at 56°C. The supernatant (400–450 µl) was transferred to a new 1.5 ml tube after 10 min centrifugation at 13,200 RPM, and genomic DNA was extracted using a silica-gel method. For blood samples red blood cells were removed with ACK lysing buffer (NH4Cl 8.29 g/L, KHCO3 1.00 g/L, EDTA 0.037 g/L, pH 7.4) before digestion with DNA lysing buffer.

#### HANDS-Nested DNA PCR

HANDS-Nested DNA PCR, a technique that combines HANDS (Homo-Tag Assisted Non-Dimer System) PCR [25], [26] with Nested PCR [27], was used to reduce primer-dimer formation and amplify genes of low copy numbers. The genomic *neo^r^* gene was first amplified using HANDS-PCR followed by Nested-PCR. The primary PCR was performed for 45 cycles followed by 25 cycles of secondary PCR with the primary PCR product as templates in a 1∶2500 dilution (final). All reactions are in a 20 µl of volume. In the primary HANDS-PCR two hybrid primers and one Tag primer were used: 5′-**CGTACTAGCGTACCACGTGTCGACT**ATTCGGCTATGACTGGGCACAACA -3′(T1-Neo-forward) at 0.02 µM, 5′-**GCGTACTAGCGTACCACGTGTCGACT**GTCAAGAAGGCGATAGAAGGCGAT-3′(T1-Neo-reverse) at 0.02 µM, and 5′-**GCGTACTAGCGTACCACGTGTCGACT**-3′ (T1) at 0.25 µM. The following touchdown thermal conditions were used : 95°C for 5 min, 5 cycles of 94°C for 30 s, 70°C for 1 min, touchdown −1°C/cycle, and 72°C for 1 min; 5 cycles of 94°C for 30 s, 68°C for 1 min, touchdown −1°C/cycle, and 72°C for 1 min; 35 cycles of 94°C for 30 s, 60°C for 1 min, and 72°C for 1 min; 72°C for 15 min at the final extension. The expected size of amplicon was 792bp. The primary PCR products were diluted with 1×Tris buffer (pH 8.0) and used as templates (1∶1250, final) in nested-PCR using the nested-primer 5′-TGAATGAACTGCAGGACGAGGCA-3′ (forward, 0.25 µM) and 5′-GGGTAGCCAACGCTATGTCCTGATA-3′ (reverse, 0.25 µM). The PCR conditions were: 95°C for 5 min, 10 cycles of 94°C for 30 s, 65°C for 1 min, touchdown −1°C/cycle, 15 cycles of 94°C for 30 s, 50°C for 1 min, and 72°C for 1 min; 72°C for 10 min at the final extension step. All the nested-PCR products were separated on a 1.5% agarose gel at the 5 v/cm for 60 min. The expected PCR products were 507 bp. House-keeping gene 18SrRNA (Supplementary Table S2) was amplified using the primary PCR thermal conditions, and 10 µl were loaded as an internal loading control. PCR Master Mix (Promega, Cat No. M7502) was used in all the reactions.

#### RNA interference

pCSCs cells were transfected with *mili*-specific small interference (si) RNA (UCGUACCUACCGAAUCGAU) or scramble siRNA (CACGUGAGGAUCACCAUCA) using an siRNA transfection kit, as instructed by the manufacturer (Qiagen). For the effect of *mili* siRNA on cell expansion, a low density of transfected cells (100/well) were seeded and counted at indicated times. For RT-PCR analysis of *mili* gene expression, a high density of transfected cells (1×10^6^/well) were seeded and harvested at 48 hrs of culture.

#### Construction of lentiviral GFP-Mili vectors

To construct the *mili* lentiviral expression vector, two primers, under the control of CMV promoter, were used to amplify the cDNA coding for *mili* gene by PCR: 5′ - GATTCGGAATTCACAACCAT GGATCCTGTCAGGCCGTTGTTCA - 3′ and 5′–TTAACGTGAATTCTTACAGG AAGAACAGGTTCCCACACAGCTG - 3′. The full-size of *mili* cDNA in plasmid pcDNA-FLAG (a gift from Dr. Nakano, Osaka University, Japan) was used as the template [15]. The synthesized *mili* cDNA from PCR, which has an *Eco*RI site in both ends derived from primers, was cloned into the lentiviral vector pLenti6-ZsGreen (Fig. 7a), which is derived from a recombination of the Zoanthus sp. green fluorescent protein (ZsGreen) (Clontech) and pLenti6/V5-TOPO (Invitrogen), at the *Eco*RI site to produce pLenti6-ZsGreen-Mili. The insert of *mili* was verified by sequencing. Pseudotype pLenti6-ZsGreen-Mili (Lenti-GFP-Mili) and pLenti6-ZsGreen viruses (Lenti-GFP) were produced by transfecting the 293FT cell line, as instructed by manufacturer (Invitrogen). The viral supernatants were harvested on 72 hr post transfection.

#### Transduction of BM cells

The BM cells, isolated from femoral and tibia bones of B6 mice, were transduced with Lenti-GFP-Mili or Lenti-GFP viruses. Briefly, BM cells (1×10^6^) were incubated with 0.3 ml viral supernatant at 37°C for 30∼60 min. Then 0.7 ml serum-free medium HBCM-2 containing 5% XLCM^TM^ [53], [64], which can expand hematopoietic progenitor cells (our unpublished data), were added into the cultures. The GFP^+^ colonies were counted as an indicator of BM cell proliferation.

#### Karyotype analysis

Exponentially growing cells were fixed using standard laboratory procedures. The cell suspension was dropped onto pre-cleaned, warm, wet, slides. The slides were aged at 90° C for 1 hour, banded with trypsin, and stained with Wright stain. Banded metaphases were analyzed using a Zeiss Axioskop 40. For each cell line, 10 metaphases were karyotyped using an Applied Imaging Karyotyping System.

To monitor genetic alterations of the pCSCs progressing to cancer, 2C4, 3B5C, 3B6C cells (5×10^6^) were injected s.c. into SCID mice, and the resultant tumors were harvested and single cells were prepared. The single cells were cultured *in vitro* until they grew sufficiently for karyotype analysis.

#### Flow cytometry

Single cells prepared from blood and tumors were stained with corresponding mAbs and then analyzed by flow cytometry, as described previously [14].

#### Histology

The tumors and tissue samples were fixed in formalin and embedded in paraffin for pathological and immunohistochemical analysis. Sections were stained by H. & E. for pathological analysis. To identify neomycin in tissues, the sections were immunostained with a polyclonal rabbit anti-neomycin (Upstate Cell Signaling Solutions, Lake Placid, NY) and followed by a horseradish peroxidase-conjugated goat anti-rabbit IgG. Sections were counterstained with haematoxylin.

#### Statistical analysis

Multiple groups of observations were analyzed by the one-way analysis of variance (ANOVA), and two groups of observations were compared by student-T test. A value of p≤0.05 was considered significant. Data are represented as mean±SD. *, p≤0.05; **, p≤0.01.

## Supporting Information

Figure S1 Incomplete differentiation of pCSCs in the CFC assay. The cells (2C4, 3B5C or 3B6C) were plated (100 or 200 cells/well) in semisolid methylcellulose medium of MethoCultTM GF M3434 (StemCell Technologies Inc. Canada) for CFC assay. The colonies were counted 2 wks after culture (A & B). The lineage-specific gene expression was analyzed by RT-PCR before or at day 11 of culture, and the BM cells were used as a positive control (C). The experiments were repeated 3 times with similar results. The data shown in A are expressed as mean±SD.
(0.20 MB TIF)

Figure S2 The effect of cytokines on pCSC differentiation in vitro. A & B, The effect of G-CSF on pCSC differentiation: The cells (75,000/flask) of 2C4, 3B5C and 3B6C clones were cultured in 10 ml R10F medium containing 10% of G-CSF-supernatant. Starting from d 5 of culture, the medium was replenished every other day with 30 ml of medium containing 10% of G-CSF supernatant. The viable cells were counted every other day until all of them died (A). The cytological alterations of the pCSCs were monitored by Wright-Giemsa staining at each time point. The micrographs (B) show a representative from the clone 3B6C of three experiments. Control cultures in the absence of G-CSF supernatant did not cause cell death (data not shown). C, The effect of GM-CSF on pCSC differentiation: 2C4 cells were cultured (100 cells/well) in R10F containing 5 ng/ml recombinant murine GM-CSF (PeproTech, Inc, Rocky Hill, NJ) in 24-well plates. The data shown are representative from the cultures in the absence (left panel) or presence of GM-CSF (right panel) of three experiments. D & E, The effect of IL-7 and IL-15 on pCSC differentiation: 2C4 cells (100/well) were cultured in the presence of IL-7 (50 ng/ml) or IL-15 (50 ng/ml) or in a combination of them. The cells were harvested on days 9 and 12 of culture and either stained with mAbs to NK1.1 and B220 (D) or cytospined for Wright-Giemsa staining (E). The data represent three experiments.
(0.48 MB TIF)

Figure S3 pCSCs can repopulate in various organs of recipients. 2C4 cells (5×10^5^) were transplanted into lethally irradiated CD45.1 B6 mice, along with 2×10^5^ recipient-type BM cells. The mice were sacrificed 5 month latter, and various organs were harvested for analysis of pCSC-derived neor gene, using HANDS-Nested DNA PCR. The data were from one of 3 experiments. The organs from control (ctrl) mice were used as the negative control, and 2C4 and 2C4G2 cell lines were used as positive controls.
(0.31 MB TIF)

Figure S4 Generation of stable eGFP expressing cell lines. 2C4 cells were transduced with Lenti-GFP viral vectors and selected in the presence of puromycin for >2 months. The drug-resistant cells were cloned by limiting dilution, and eGFP^+^ clones were identified by flow cytometry. The histogram depicted the fluorescent intensity of a representative clone 2C4G2, which was used throughout the experiments.
(0.05 MB TIF)

Figure S5 pCSC-derived metastatic tumors in various organs. A, metastatic tumor in the spleen, liver, pancreas and prostates. The data represent tissues derived from the mice injected with 2C4 (spleen and liver) or 3B5C (pancreas and prostate). Original magnification: ×400.
(1.64 MB TIF)

Figure S6 Restrained tumorigenesis of pCSCs after intravenous inoculation. SCID mice were injected i.v. with 5×10^5^ 2C4, 3B5C or 3B6C (n = 3/group). As a control, the lethally irradiated B6 mice were injected i.v. with the same number of 2C4, 3B5C or 3B6C cells (n = 4/group) together with 5×10^5^ recipient-type BM cells. The mice were sacrificed 5 months later, and various organs or tissues, including the spleen, liver, kidney, lungs, intestines, pancreas and blood, were harvested from the SICD and BM-reconstituted B6 mice for pathological examination. None of the organs developed cancer, except for the spleens of SCID mice. A, The structure of normal spleen of SCID mice; B, The leukemic alteration in the spleen of SCID mice injected i.v. with pCSCs: the micrograph shown is from a mouse injected i.v. with 36BC cells; C, Blast cells detected in the blood smears: a representative from a SCID mouse injected with 2C4 cells; D, Normal appearance of the spleens from the BM-reconstituted mice: the micrograph shows a representative from a mouse injected with pCSCs (2C4 clone). Original magnification for H& E. staining sections: ×400; blood smear: ×1000. The insets are enlargements indicated by arrows.
(1.29 MB TIF)

Table S1 Effect of environments on the tumorigenesis of pCSCs
(0.04 MB DOC)

Table S2 Primer sequence used for RT-PCR
(0.13 MB DOC)
