# Supplementary figures and images for: Precancerous Stem Cells Have the Potential for both Benign and Malignant Differentiation
Source: PLoS One. 2007 Mar 14;2(3):e293. doi: 10.1371/journal.pone.0000293 (PMC1808425; doi:10.1371/journal.pone.0000293)

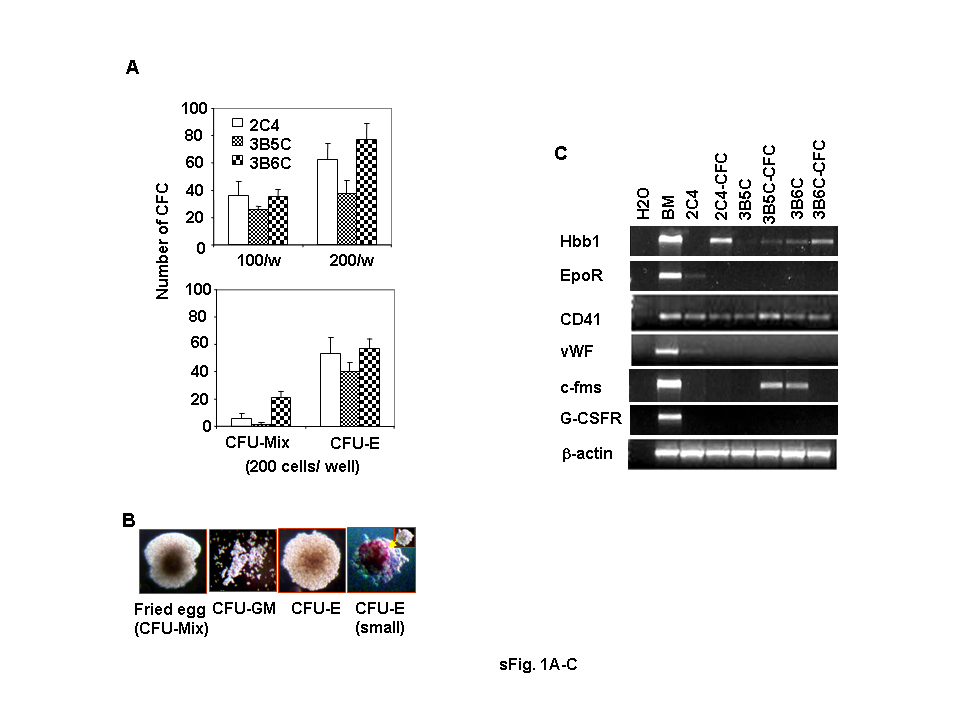

Supplement: Figure S1 — Incomplete differentiation of pCSCs in the CFC assay. The cells (2C4, 3B5C or 3B6C) were plated (100 or 200 cells/well) in semisolid methylcellulose medium of MethoCultTM GF M3434 (StemCell Technologies Inc. Canada) for CFC assay. The colonies were counted 2 wks after culture (A & B). The lineage-specific gene expression was analyzed by RT-PCR before or at day 11 of culture, and the BM cells were used as a positive control (C). The experiments were repeated 3 times with similar results. The data shown in A are expressed as mean±SD. (0.20 MB TIF) [file pone.0000293.s001.tif]

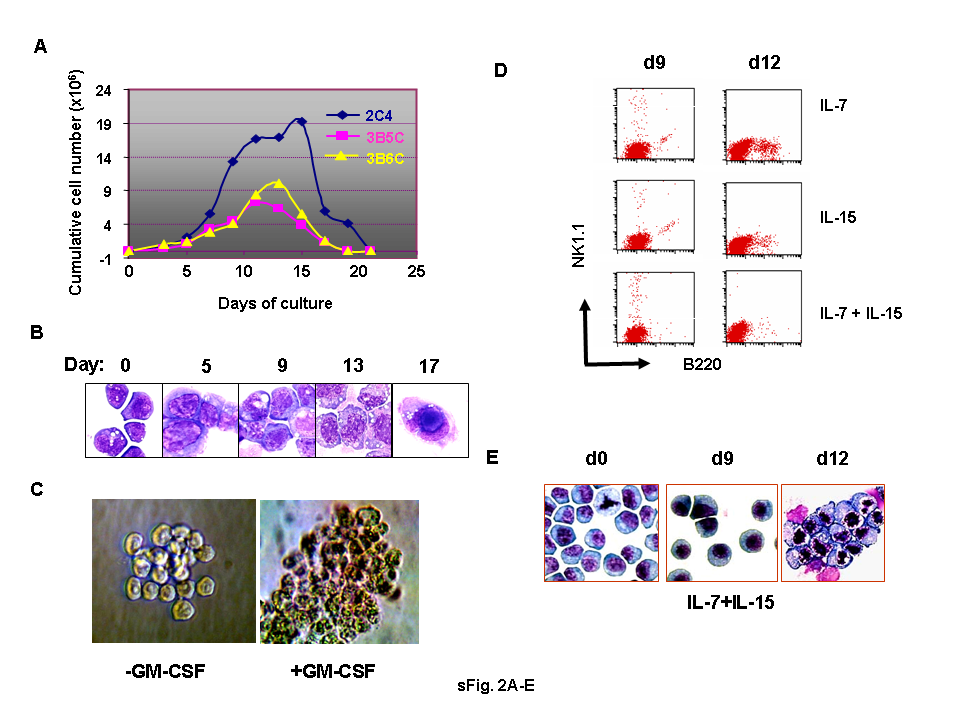

Supplement: Figure S2 — The effect of cytokines on pCSC differentiation in vitro. A & B, The effect of G-CSF on pCSC differentiation: The cells (75,000/flask) of 2C4, 3B5C and 3B6C clones were cultured in 10 ml R10F medium containing 10% of G-CSF-supernatant. Starting from d 5 of culture, the medium was replenished every other day with 30 ml of medium containing 10% of G-CSF supernatant. The viable cells were counted every other day until all of them died (A). The cytological alterations of the pCSCs were monitored by Wright-Giemsa staining at each time point. The micrographs (B) show a representative from the clone 3B6C of three experiments. Control cultures in the absence of G-CSF supernatant did not cause cell death (data not shown). C, The effect of GM-CSF on pCSC differentiation: 2C4 cells were cultured (100 cells/well) in R10F containing 5 ng/ml recombinant murine GM-CSF (PeproTech, Inc, Rocky Hill, NJ) in 24-well plates. The data shown are representative from the cultures in the absence (left panel) or presence of GM-CSF (right panel) of three experiments. D & E, The effect of IL-7 and IL-15 on pCSC differentiation: 2C4 cells (100/well) were cultured in the presence of IL-7 (50 ng/ml) or IL-15 (50 ng/ml) or in a combination of them. The cells were harvested on days 9 and 12 of culture and either stained with mAbs to NK1.1 and B220 (D) or cytospined for Wright-Giemsa staining (E). The data represent three experiments. (0.48 MB TIF) [file pone.0000293.s002.tif]

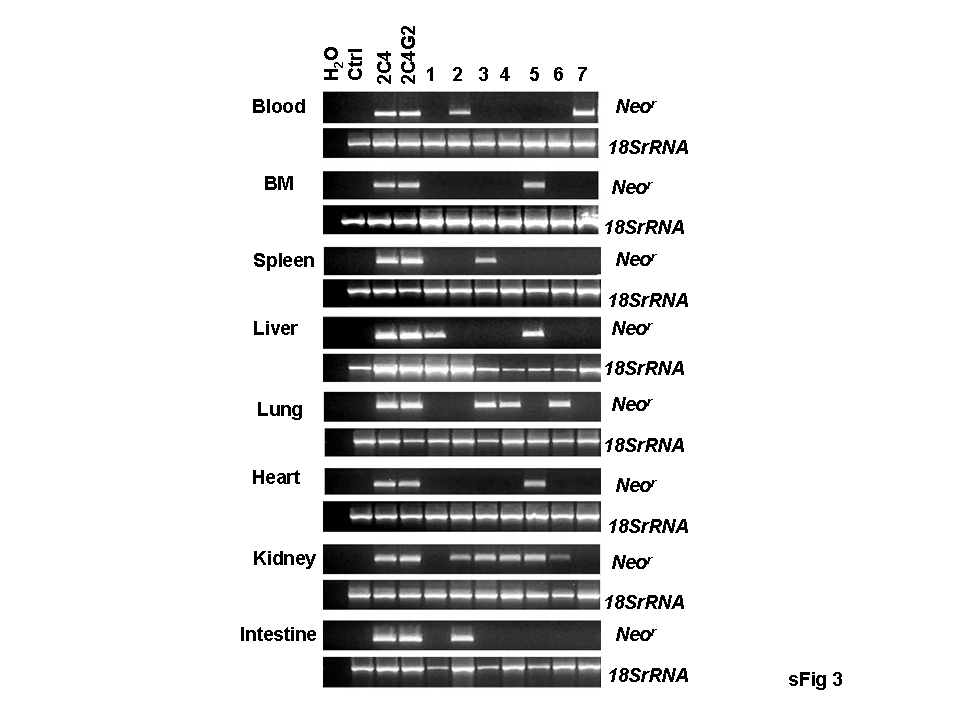

Supplement: Figure S3 — pCSCs can repopulate in various organs of recipients. 2C4 cells (5×10^5^) were transplanted into lethally irradiated CD45.1 B6 mice, along with 2×10^5^ recipient-type BM cells. The mice were sacrificed 5 month latter, and various organs were harvested for analysis of pCSC-derived neor gene, using HANDS-Nested DNA PCR. The data were from one of 3 experiments. The organs from control (ctrl) mice were used as the negative control, and 2C4 and 2C4G2 cell lines were used as positive controls. (0.31 MB TIF) [file pone.0000293.s003.tif]

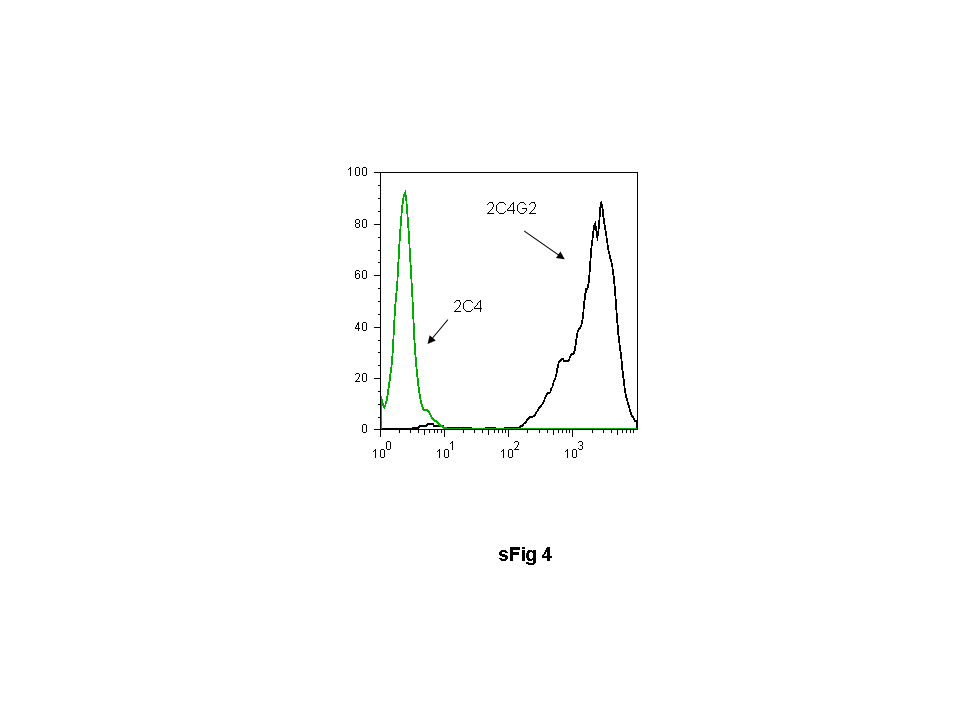

Supplement: Figure S4 — Generation of stable eGFP expressing cell lines. 2C4 cells were transduced with Lenti-GFP viral vectors and selected in the presence of puromycin for >2 months. The drug-resistant cells were cloned by limiting dilution, and eGFP+ clones were identified by flow cytometry. The histogram depicted the fluorescent intensity of a representative clone 2C4G2, which was used throughout the experiments. (0.05 MB TIF) [file pone.0000293.s004.tif]

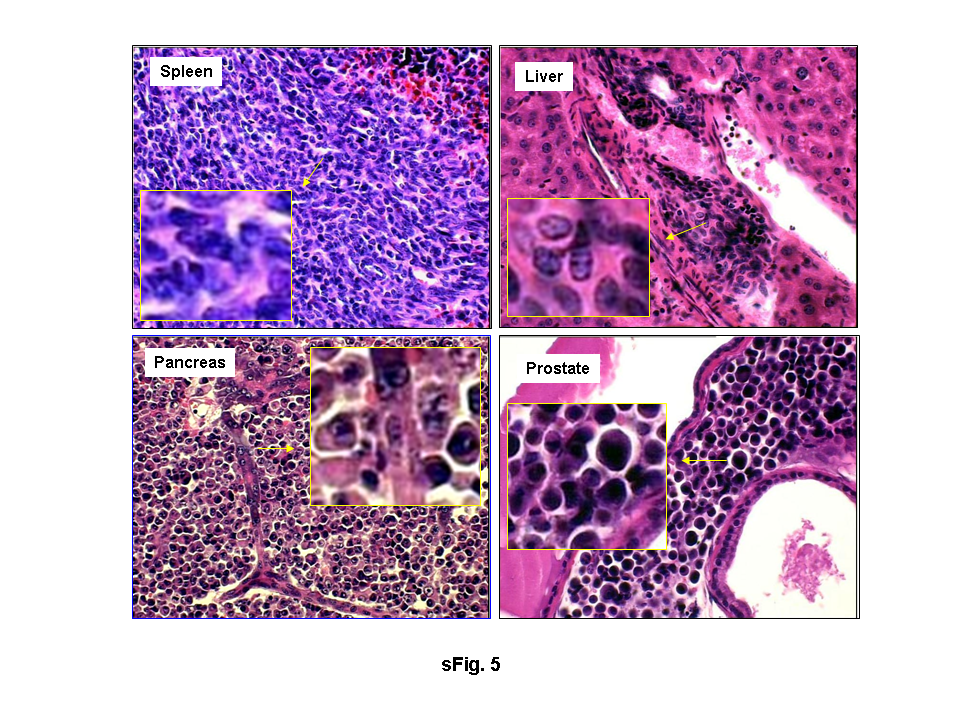

Supplement: Figure S5 — pCSC-derived metastatic tumors in various organs. A, metastatic tumor in the spleen, liver, pancreas and prostates. The data represent tissues derived from the mice injected with 2C4 (spleen and liver) or 3B5C (pancreas and prostate). Original magnification: ×400. (1.64 MB TIF) [file pone.0000293.s005.tif]

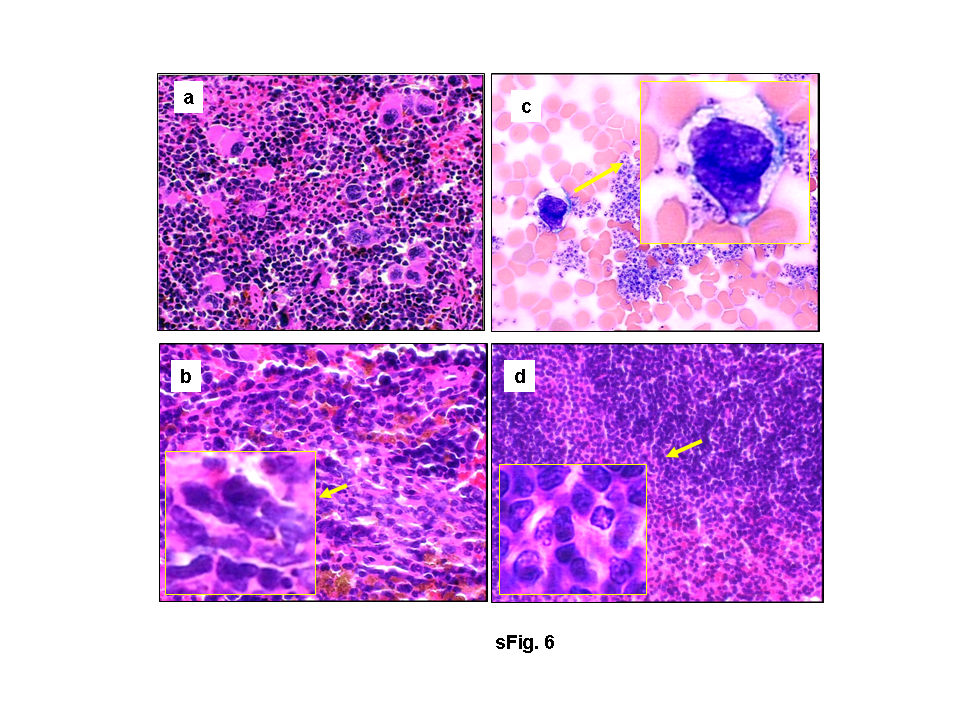

Supplement: Figure S6 — Restrained tumorigenesis of pCSCs after intravenous inoculation. SCID mice were injected i.v. with 5×10^5^ 2C4, 3B5C or 3B6C (n = 3/group). As a control, the lethally irradiated B6 mice were injected i.v. with the same number of 2C4, 3B5C or 3B6C cells (n = 4/group) together with 5×10^5^ recipient-type BM cells. The mice were sacrificed 5 months later, and various organs or tissues, including the spleen, liver, kidney, lungs, intestines, pancreas and blood, were harvested from the SICD and BM-reconstituted B6 mice for pathological examination. None of the organs developed cancer, except for the spleens of SCID mice. A, The structure of normal spleen of SCID mice; B, The leukemic alteration in the spleen of SCID mice injected i.v. with pCSCs: the micrograph shown is from a mouse injected i.v. with 36BC cells; C, Blast cells detected in the blood smears: a representative from a SCID mouse injected with 2C4 cells; D, Normal appearance of the spleens from the BM-reconstituted mice: the micrograph shows a representative from a mouse injected with pCSCs (2C4 clone). Original magnification for H& E. staining sections: ×400; blood smear: ×1000. The insets are enlargements indicated by arrows. (1.29 MB TIF) [file pone.0000293.s006.tif]
