# Supplementary material for: Precancerous Stem Cells Have the Potential for both Benign and Malignant Differentiation
Source: PLoS One. 2007 Mar 14;2(3):e293. doi: 10.1371/journal.pone.0000293 (PMC1808425; doi:10.1371/journal.pone.0000293)
Supplement: Table S1 — Effect of environments on the tumorigenesis of pCSCs (0.04 MB DOC) [file pone.0000293.s007.doc]

**Supplementary Table 1: Effect of environments on the tumorigenesis of pCSCs**

| **Mice** | **s.c.**^5^ | | | **i.p.** | | | **i.v.** | | |
| --- | --- | --- | --- | --- | --- | --- | --- | --- | --- |
|  | 2C4 | 3B5C | 3B6C | 2C4 | 3B5C | 3B6C | 2C4 | 3B5C | 3B6C |
| Scid mice^1^ | Solid  (n=19) | Solid  (n=5) | Leukemic  (n= 5) | Solid  (n=19) | Solid  (n=5) | Leukemic  (n=5) | Leukemic  (n=5) | Leukemic  (n=5) | Leukemic  (n=5) |
| BM-reconstituted B6 mice^2^ | Solid  (n=3) | None^6^  (n=3) | None  (n=3) | Solid  (n=3) | None  (n=3) | None  (n=3) | None  (n=20) | None  (n=10) | None  (n=10) |
| B6 mice^3^ | None^4^  (n=15) | Non  (n=13) | None  (n=13) | None  (n=15) | None  (n=13) | None  (n=13) | None  (n=15) | None  (n=15) | None  (n=15) |

1. SCID mice were injected either s.c. or i.p. with 5 x 10^6^ pCSCs or i.v. with 5 x 10^5^ pCSCs.
2. Lethally irradiated, BM-reconstituted mice were injected either s.c. or i.p. with 5 x 10^6^ pCSCs or i.v. with 2 x 10^6^ pCSCs, along with 5 x 10^5^ recipient-type of BM cells. 2C4 cells developed solid tumors in situ within 3 wks of i.p. or s.c. inoculation, whereas a sole 3B5C tumor in situ was eliminated after being palpable.
3. B6 mice were injected with 5 x 10^6^ pCSCs and monitored more than 5 months.
4. None: Mice did not develop solid or leukemic tumors; solid: progressive tumors in situ.
5. The SCID mice injected s.c. or i.p. were observed until they developed tumors and sacrificed, and the mice injected i.v. were sacrificed 5 months or later after injection with no overt clinical symptoms. Leukemia was verified by microscopic examination of spleens. The number shown is the total numbers of mice used for experiments. The percentage of tumor incidence was variable with experiments.
6. One mouse developed a 3 x 3 tumor, which shrunk in a few days.
