# Supplementary material for: Precancerous Stem Cells Have the Potential for both Benign and Malignant Differentiation
Source: PLoS One. 2007 Mar 14;2(3):e293. doi: 10.1371/journal.pone.0000293 (PMC1808425; doi:10.1371/journal.pone.0000293)
Supplement: Table S2 — Primer sequence used for RT-PCR (0.13 MB DOC) [file pone.0000293.s008.doc]

**Supplementary Table 2: Primer sequence used for RT-PCR**

**Gene Gene bank No Sequence Products**

| 1. EPOR-For | NM_010149 | 5'- GGACACCTACTTGGTATTGG - 3' | 452bp |
| --- | --- | --- | --- |
| EPOR-Rev |  | 5'- GACGTTGTAGGCTGGAGTCC - 3' |  |
|  |  |  |  |
| 2. cfms-For | X06368 | 5' - CTGGAGAAGAAATATGTGCG - 3' | 448bp |
| cfms-Rev |  | 5' - TTCAGACCAAGCGAGAAGAT - 3' |  |
|  |  |  |  |
| 3. GCSFR-For | NM_007782 | 5' - TCATCACTCTGCCTCACTTG - 3' | 472bp |
| GCSFR-Rev |  | 5' - GAGACTACATCAGGGCCAAT - 3' |  |
|  |  |  |  |
| 4. CD41-For | NM_010575 | 5' - ATGGTTACTGTGCAGGTCATGCTG - 3' | 484bp |
| CD41-Rev |  | 5' - ATGGCGGATATCACTGTCAAGCTC - 3' |  |
|  |  |  |  |
| 5. Hbb1-For | XM_489729 | 5' - CATAGTTGTGTTGACTCACAACCC - 3' | 509bp |
| Hbb1-Rev |  | 5' - TTCACAGGCAAGAGCAGGAAAG - 3' |  |
|  |  |  |  |
| 6. vWF-For | NM_011708 | 5' - GCTTCCAACTGAACTGTGAGACCT - 3' | 509bp |
| vWF-Rev |  | 5' -GGCTGTGATGTCTTTGCAATCAGG - 3' |  |

| 7. | POUF1/Oct-4-For | | NM_013633 | 5'-AGCACGAGTGGAAAGCAACTCAGA -3' | | | 497 bp | | | | |  | | | |
| --- | --- | --- | --- | --- | --- | --- | --- | --- | --- | --- | --- | --- | --- | --- | --- |
|  | POUF1/Oct-4-Rev | | | | | 5'-CAAGCTGATTGGCGATGTGAGTGA-3' | |  | | | | | |  | |
| 8. | TDGF1/Cripto-For | NM_011562 | | | | 5'-AGGACAGACAGGCCTACACAGAAA-3' | | 498 bp | | | | | |  | |
|  | TDGF1/Cripto-For |  | | | | 5'-TGTGAGGGTCTTGCCATTCAGTCT -3' | |  | | | | | |  | |
|  |  |  | | | |  | |  | | | | | |  | |
| 9. | Zfp42/REX1-For | NM_009556 | | | | 5'-AACTCCTAGCCGCCTAGATTTCCACT -3' | | 492 bp | | | | | |  | |
|  | Zfp42/REX1-Rev |  | | | | 5'-ACTCTGGTATTCTGGACTGGCCTT-3' | |  | | | | | |  | |
|  |  |  | | | |  | |  | | | | | |  | |
| 10. | Fzd2-For | NM_020510 | | | | 5'-ACATCGCCTACAACCAGACCATCA-3' | | 494 bp | | | | | |  | |
|  | Fzd2-Rev |  | | | | 5'-ATAGCTGAGATAGGACGGCACCTT-3' | |  | | | | | |  | |
|  |  |  | | | |  | |  | | | | | |  | |
| 11. | Fzd5-For | NM_022721 | | | | 5'-CCACAGGTACCTAGCTTGTCGTTA -3' | | 648 bp | | | | | |  | |
|  | Fzd5-Rev |  | | | | 5'-ACAGATGGGCGTGTACATAGAGCA-3' | |  | | | | | |  | |
|  |  |  | | | |  | |  | | | | | |  | |
| 12. | Catnb-For | NM_007614 | | | | 5'-TCTACGCCATCACGACACTGCATA -3' | | 507bp | | | | | |  | |
|  | Catnb-Rev |  | | | | 5'-CAGCTGCACAGGTGACCACATTTA-3' | |  | | | | | |  | |
|  |  |  | | | |  | |  | | | | | |  | |
| 13. | Smo-For | NM_176996 | | | | 5'-CCCAATTGGCCTGGTGCTTATTGT -3' | | 497bp | | | | | |  | |
|  | Smo-Rev |  | | | | 5'-CCTTGGCGATCATCTTGCTCTTCT-3' | |  | | | | | |  | |
|  |  |  | | | |  | |  | | | | | |  | |
| 14. | Abcg2-For | NM_011920 | | | | 5'-TTCTGTCACCAGCTCCGATGGATT -3' | | 492bp | | | | | |  | |
|  | Abcg2-Rev |  | | | | 5'-TACAGACACCACACTTTGGCCTGT-3' | |  | | | | | |  | |
|  |  |  | | | |  | |  | | | | | |  | |
| 15. | Flt3-For | NM_010229 | | | | 5'-TGCCTGGTTCACGAGAAGTTCAGT -3' | | 490bp | | | | | |  | |
|  | Flt3-Rev |  | | | | 5'-AAGTTAGCGTCGACAGGAATGCCA-3' | |  | | | | | |  | |
|  |  |  | | | |  | |  | | | | | |  | |
| 16. | beta-Actin-For | NM_007393 | | | | 5'-CAGCTTCTTTGCAGCTCCTTCGTT-3' | | 1137 bp | | | | | |  | |
|  | beta-Actin-Rev |  | | | | 5'-TACTCCTGCTTGCTGATCCACATC-3' | |  | | | | | |  | |
|  |  |  | | | |  | | | |  | | | | |  |
| 17. | c-Myc-For | NM_010849 | | | | 5'-AAAGACAGCACCAGCCTGAGC-3' | | | 492 bp | | | |  | | |
|  | c-Myc-Rev |  | | | | 5'-AGCTGGATAGTCCTTCCTTGTGGA-3' | | |  | | | |  | | |
|  |  |  | | | |  | | |  | | | |  | | |
| 18. | Notch1-For | NM_008714 | | | | 5'-TTGAGATGCTCCCAGCCAAGT -3' | | | 494 bp | | | |  | | |
|  | Notch1-Rev |  | | | | 5'-GGGTTCTGGCTGCACTCATTAACA-3' | | |  | | | |  | | |
|  |  |  | | | |  | | |  | | | |  | | |
| 19. | Bcl2-For | NM_009741 | | | | 5'-TGCCAGGACGTCTCCTCTCAG-3’ | | | 501bp | | | |  | | |
|  | Bcl2-Rev |  | | | | 5'-AGGTATGCACCCAGAGTGATGCAG-3’ | | |  | | | |  | | |
|  |  |  | | | |  | | |  | | | |  | | |
| 20. | Nanog-For | AY455285 | | | | 5' - AAGATGCGGACTGTGTTCTCTCAG - 3' | | | 490 bp | | | |  | | |
|  | Nanog-Rev |  | | | | 5' - GTACGTAAGGCTGCAGAAAGTCCT - 3' | | |  | | | |  | | |
|  |  |  | | | |  | | |  | | | |  | | |
| 21. | STAT3-For | NM_011486 | | | | 5' - CTTCACTAAGCCGCCAATTGGAAC - 3' | | | 538bp | | | |  | | |
|  | STAT3-Rev |  | | | | 5' - GACAAGTGGAGACACCAGGATGTT- 3' | | |  | | | |  | | |
|  |  |  | | | |  | | |  | | | |  | | |
| 22. | Endoglin-For | NM_007932 | | | | 5' - GTATCACCTTTGGTGCCTTCCTGA - 3' | | | 633 bp | | | |  | | |
|  | Endoglin-Rev |  | | | | 5' - TAAGCTCCCTCAGCTTCTGTTTCC - 3' | | |  | | | |  | | |
|  |  |  | | | |  | | | |  | | | | |  |
| 23. | Bmi-1-For | M64067 | | | | 5' - AGCAGCAATGACTGTGATGCACTTGAG - 3' | | | | | 245 bp | | | | |
|  | Bmi-1-Rev |  | | | | 5' - GCTCTCCAGCATTCGTCAGTCCATCCC - 3' | | | | |  | | | | |
|  |  |  | | | |  | | | | |  | | | | |
| 24. | Mili/Piwil2-For | NM_021308 | | | | 5'-TGAACTGTAAACTGGGTGGTGAGC -3' | | | | | 497 bp | | | | |
|  | Mili/Piwil2-Rev |  | | | | 5'-CAC TCA CAG CTG GTT ATG GTA TGA -3' | | | | |  | | | | |
|  |  |  | | | |  | | | | |  | | | | |
| 25. | Miwi/Piwil1-For | AF438405 | | | | 5' - GCTTGGAGTGGCTGCAATGAATAC - 3' | | | | | 500 bp | | | | |
|  | Miwi/Piwil1-Rev |  | | | | 5' - TTTGGCTCTCTGTGGATGCTCT - 3' | | | | |  | | | | |
|  |  |  | | | |  | | | |  | | | | |  |
| 26. 18sRNA-For | | | X00686 | | 5'-TAACGAGGATCCATTGGAGGGCAA -3' | | | | | | 1095bp | | | | |
| 18sRNA-Rev | | |  | | 5'-AATCAACGCAAGCTTATGACCCGC-3' | | | | | |  | | | | |
